# Spontaneous evolution of equilibrium morphology in phospholipid-cholesterol monolayers

**DOI:** 10.1126/sciadv.abl9152

**Published:** 2022-04-06

**Authors:** Cain Valtierrez-Gaytan, Joseph M. Barakat, Mitchell Kohler, Khanh Kieu, Benjamin L. Stottrup, Joseph A. Zasadzinski

**Affiliations:** 1 Department of Chemical Engineering and Materials Science, University of Minnesota, Minneapolis, MN 55455, USA.; 2 Department of Chemical Engineering, University of California, Santa Barbara, Santa Barbara, CA 93106, USA.; 3 Department of Physics, Augsburg University, Minneapolis, MN 55454, USA.

## Abstract

Competition between intradomain electrostatic repulsions and interdomain line tension leads to domain shape transitions in phase-separating lipid monolayers. The question remains if these morphologies are energy minima or are kinetically trapped metastable states. We show the reversible evolution of uniform width stripe domains from polydisperse semicircular domains in monolayers of dipalmitoylphosphatidylcholine (DPPC), hexadecanol (HD) or palmitic acid (PA), and dihydrocholesterol (DChol). The initial semicircular domains grow at a fixed 2:1 DPPC:HD (or PA) stoichiometry, depleting the liquid phase of HD, leaving behind a liquid enriched in DPPC and DChol. At higher surface pressures, the remaining DPPC precipitates onto existing domains, decreasing the ratio of line tension to the square of the dipole density difference, λ/μ^2^. Theory predicts that, as λ/μ^2^ decreases, circular domains reversibly transform to uniform width stripes as the minimum energy structure. Measuring the stripe width provides the first estimates of λ/μ^2^ at liquid condensed–liquid expanded phase coexistence.

## Teaser

Monolayer domains reversibly convert from polydisperse circles to uniform width stripes due to changing boundary composition.

## INTRODUCTION

**Fig. 1.**
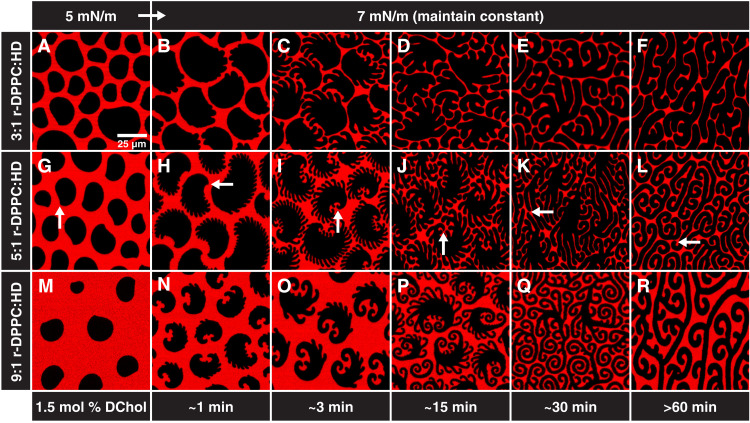
Circle-to-stripe transition at constant surface pressure in phospholipid-cholesterol monolayers. Representative confocal microscopy images of monolayers spread from 3:1 (**A** to **F**), 5:1 (**G** to **L**), or 9:1 (**M** to **R**) r-DPPC:HD mole ratio with 1.5 mol % DChol at 22°C. (A, G, and M) (first column) At low surface pressures (π > 6 mN/m), semicircular domains form with a cusp (white arrow). (B, H, and N) (second column) Uniformly spaced fingers develop opposite the cusps when compressed to a surface pressure of 7 mN/m. (C, I, and O) (third column) Holding the surface pressure constant for 3 min results in the fingers growing. (D, J, and P) (fourth column) After roughly 15 min, fingers continue to grow and begin to coalesce, making fewer and wider fingers. (E, K, and Q) (fifth column) After 30 min, the finger growth continues at the expense of the core of the domain. (F, L, and R) (sixth column) Waiting longer than 60 min results in extended stripes of uniform width but variable length. The arrows in the second row show that each domain has a single cusp that is retained throughout the transition. The average stripe widths in (F), (L), and (R) are 6.0 ± 0.3 μm, 4.1 ± 0.2 μm, and 4.0 ± 0.2 μm, respectively.

Surfactant monolayers are ubiquitous in nature and daily life, stabilizing liquid emulsion drops and foam bubbles, maintaining low surface tension in the lung alveoli (*1*, *2*), and stabilizing tear films in the eye (*3*). Fluorescence optical microscopy (*4*, *5*), synchrotron x-ray diffraction and reflectivity (*6*, *7*), and atomic force microscopy (*8*, *9*) have provided ample evidence of phase separation into immiscible liquid ordered–liquid disordered (L_o_-L_d_) or liquid condensed–liquid expanded (L_C_-L_E_) domains in lipid and cholesterol monolayers at the air-water interface (Fig. 1 and fig. S1). Here, we show reversible circle-to-stripe domain shape transitions with changes in surface pressure, composition, or temperature that result from competition between dipole-dipole interactions within the domains and the line tension at the domain boundaries (*5*). Similar domain formation is key to the “raft” model of cell membranes (*10*) in which nanometer to micrometer length scale, phase-separated immiscible liquid domains or rafts with different composition can sort and compartmentalize proteins in both time and space to facilitate physiological functions (*10*). Phase separation and domain formation are also important in clinical lung surfactants (LSs) used to treat neonatal respiratory distress syndrome in premature infants (*11*).

The opposing energies of line tension, λ, at the domain boundaries and the intradomain repulsion due to differences in the dipole density between phases, μ, determine the domain shapes. λ is the energy per unit length of the monolayer domain boundaries and is the two-dimensional (2D) analog of surface tension in three dimensions (*5*, *12*, *13*). Differences in lipid composition, chain length, tilt, or local order at the domain boundaries determine the magnitude of λ (*5*, *14*). Experimental measurements of λ are typically done by analysis of spontaneous or imposed domain shape fluctuations; such analyses have been limited to immiscible liquid-liquid (L_o_-L_d_) domains as fluctuations are too small to measure at solid-liquid (L_C_-L_E_) domain boundaries. Measurements of L_o_-L_d_ line tension are on the order of 0.1 to 10 pN (picojoules/meter) (*13*–*17*). The line tension acts to minimize the ratio of domain perimeter to domain area, just as surface tension minimizes the surface area–to-volume ratio in three dimensions.

The head-tail asymmetry of surface-active molecules at an air-water (or oil-water) interface leads to a net dipole moment in the direction normal to the interface (*5*, *18*). The magnitude of the dipole moment is invariably different in coexisting monolayer phases because of differences in composition, molecular packing, or tilt orientation, resulting in a difference in the average dipole density, μ (*5*, *18*). Oriented dipoles repel each other, so high–aspect ratio domains such as stripes separate the dipoles to minimize the overall dipole-dipole energy at the cost of increasing the ratio of domain perimeter to domain area, which increases the line tension energy. The dipole contribution to the energy is greater in asymmetric monolayers than in symmetric bilayers, where the dipoles of each monolayer leaflet partially cancel the other. There are few experimental values of the dipole density difference in monolayers, but available data suggest 0.25 to 0.5 D per molecule at the interface (*19*, *20*). The dipole density difference also creates a long-range repulsion between neighboring domains that keeps domains from coalescing on experimental time scales (*5*).

Continuum theories based on the competition between the energetic contributions of dipole-dipole repulsion within domains and the line tension at the domain boundaries provide qualitative and quantitative predictions for the sizes and shapes of lipid monolayer domains (*4*, *5*, *15*, *19*, *21*–*25*). Similar models have been successfully used to describe ferrofluids (*12*) or superconductors (*26*) in an applied magnetic field. Models based on opposing energetic contributions display common features that set domain shapes and length scale from nanometers (diblock copolymers) to micrometers (lipid monolayers) to centimeters (Turing patterns and convective rolls). For lipid monolayers, the free energy per molecule, *F*/*N*, in noninteracting circular domains of radius *r* (*N* = π*r*^2^/*a_o_*, *a_o_* is the average area per molecule in the domain) is set by the opposing energetics of line tension, λ, and the square of the dipole density difference, μ^2^ (*5*)

$$\frac{F}{N}=\frac{2a_{o}}{r}[\lambda -\mu^{2}\text{ln}(\frac{4r}{e^{2}\delta })]$$ (1)

In Eq. 1, *e* is the exponential constant, 2.714 and δ~1 nm is a molecular cutoff for the intradomain dipole-dipole interactions (*5*). From Eq. 1, the minimum energy radius, *r_o_*, for noninteracting domains is (∂(*F*/*N*)/∂*r* = 0)

$$r_{o}=\frac{e^{3}\delta }{4}\text{exp}[\frac{\lambda }{\mu^{2}}]$$ (2)

The important material parameter in Eq. 2 is the Bond number, *N*_Bo_ = μ^2^/λ, which is the dimensionless ratio of the square of the dipole density difference to the line tension. Equation 2 shows that increasing μ^2^ favors smaller domains and increasing λ favors larger domains. Stability analysis of Eqs. 1 and 2 shows that circular domains with *r* ≤ *r_o_*/*e* are unstable with respect to coalescence and domains with r≥e13ro are unstable to elliptical or higher-order distortions (*5*, *25*, *27*). The Bond number appears in many different systems in which there is a competition between long-range contributions to the energetics (gravity, magnetic forces, and electrostatics) and line or surface tension (*12*).

However, over the past 40 years of visualizing monolayer domains (*28*), there have been few, if any, experimental observations of equilibrium circular domain size distributions in either L_o_-L_d_ or L_C_-L_E_ phase-separated monolayers that are quantitatively consistent with the predictions of Eq. 2 (*5*, *14*, *25*, *27*, *29*, *30*). Instead, circular domains are polydisperse and vary from experiment to experiment even for the same monolayer composition at a given surface pressure and temperature. The size distribution depends on compression rate and aging and is different for surface pressure changes at constant temperature or temperature changes at constant surface pressure. An equilibrium size distribution based on Eq. 2 should be reproducible at a fixed monolayer composition, surface pressure, and temperature and should also be independent of how those conditions are approached (*14*). Hence, it is unclear if there are fundamental limitations to the continuum theory or if there exist additional contributions to the energy yet to be understood. More likely, the kinetics of domain nucleation and growth trap monolayer domains in long-lived metastable states and the energy differences due to line tension and electrostatics are not sufficient to allow a system of circular domains to reach an equilibrium domain size distribution (Eq. 2) over experimentally accessible time scales.

Unfortunately, equilibrium theories do not provide any estimate of the rate at which the domain size distribution reaches equilibrium. In monolayers, domain coalescence is inhibited by dipole-dipole repulsion between domains. This requires equilibration by Ostwald ripening, or the “evaporation” of molecules from certain domains and “condensation” onto other domains (*31*, *32*), which does not occur over experimentally accessible time scales of days (*33*). However, the continuum theory has provided essential insight into the circle-to-stripe transitions observed near liquid-liquid miscibility critical points (*22*, *34*), as well as elliptical and higher-order domain shape evolution when domains are forced to grow by coalescing in electric fields (*27*). The continuum theory is also key to understanding the raft model of cell membranes (*10*, *35*–*37*), in which phase-separated immiscible liquid domains or rafts spontaneously form with different composition or local order that can sort and compartmentalize functional proteins in both time and space to facilitate complex tasks (*5*, *10*, *14*, *26*, *34*, *37*).

Here, we show that phase-separated ternary mixtures of dipalmitoylphosphatidylcholine (DPPC), hexadecanol [HD; or palmitic acid (PA)], and cholesterol (Fig. 1) undergo reversible changes from polydisperse semicircular domains via a Mullins-Sekerka growth instability to stripes of uniform width. The transition is consistent with a change in the boundary composition of the domains that leads to a decrease in the line tension at the domain boundary. The semicircular L_C_ domains grow at a fixed 2:1 DPPC:HD (or PA) stoichiometry at low surface pressure, which depletes the L_E_ phase of HD (or PA). At higher surface pressures, the remaining DPPC condenses onto the domains, which decreases the line tension, causing *N*_Bo_ to increase. As *N*_Bo_ increases, the minimum energy morphology changes from circular domains to uniform width stripes (*18*). The evolution to equilibrium width stripes is repeatable between experiments, and the line widths are the same if approached by changing surface pressure at constant temperature or changing the temperature at constant surface pressure. By changing the surface pressure and temperature back to the original conditions, the stripes spontaneously return to the polydisperse semicircular domain morphology. Measuring the stripe width provides the first measurements of *N*_Bo_ as a function of monolayer composition, surface pressure, and temperature at liquid condensed–liquid expanded phase coexistence.

## RESULTS

**Fig. 2.**
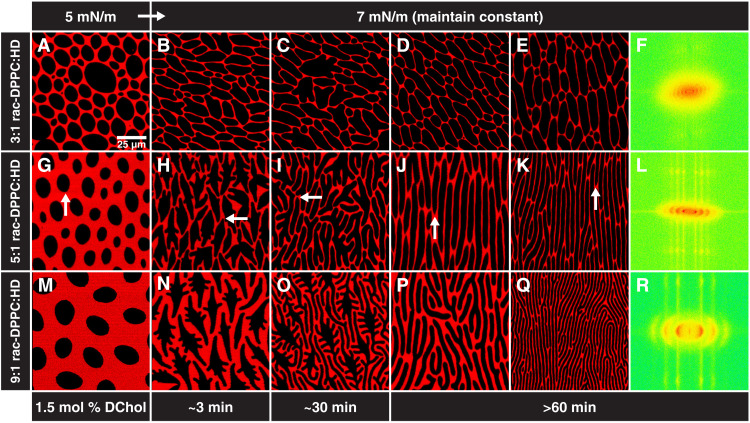
DPPC chirality determines domain chirality. Representative confocal microscopy images of films spread from a 3:1 (**A** to **E**), 5:1 (**G** to **K**), or 9:1 (**M** to **Q**) rac-DPPC:HD mole ratio with 1.5 mol % DChol at 22°C. (A, G, and M) Domains assume an elliptical shape at low surface pressures (white arrow indicates cusp). (B, H, and N) Regularly spaced, axisymmetric fingers form at 7 mN/m opposite the cusp. The intermediate morphology follows a similar progression as in the chiral r-DPPC mixture. (C, I, and O) However, here, there is less branching, and the fingers remain straight. (D, J, and P) Highly aligned stripes of uniform width are found after holding the surface pressure constant for longer than an hour. A single cusp is positioned at one end of a stripe. The stripe widths in (D), (J), and (O) are 6.5 ± 0.4 μm, 4.4 ± 0.2 μm, and 4.0 ± 0.2 μm, respectively. (E, K, and Q) Increasing the surface pressure further after the stripes have been formed causes the stripes to become more aligned and decrease in width. The stripe widths in (E), (K), and (Q) are 6.8 ± 0.4 μm, 2.9 ± 0.1 μm, and 2.0 ± 0.1 μm, respectively. (**F**, **L**, and **R**) FFT of lower-magnification images of (E), (K), and (Q) shows multiple orders of reflection and demonstrate the high orientational order of the stripes.

**Fig. 3.**
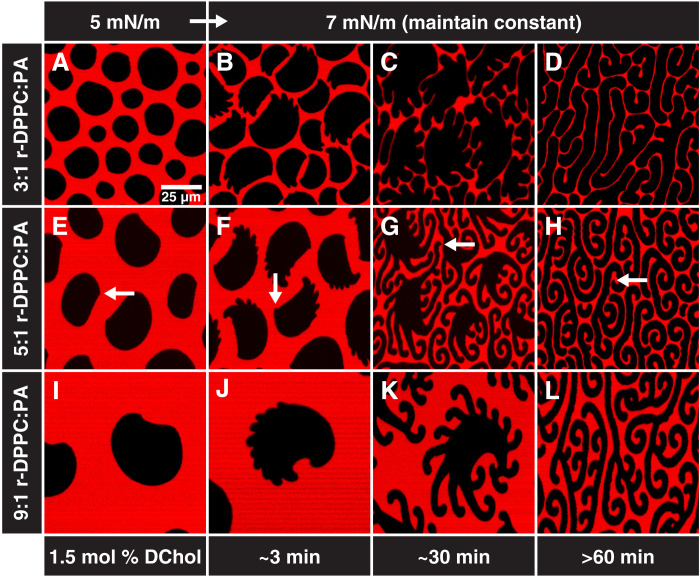
PA and HD are interchangeable. Representative confocal microscopy images of films spread from a 3:1 (**A** to **D**), 5:1 (**E** to **H**), or 9:1 (**I** to **L**) r-DPPC to PA mole ratio with 1.5 mol % DChol at 22°C. Shape transitions are similar to those observed in ternary monolayers containing HD (Fig. 1). (A, E, and I) Asymmetric, semicircular domains nucleate at low surface pressures and grow with increasing surface pressure. (B, F, and J) Increasing the surface pressure to 7 mN/m causes finger instabilities with a well-defined spacing to develop. (C, G, and K) Holding the surface pressure at 7 mN/m results in finger growth. (D, H, and L) Waiting approximately an hour at constant surface pressure results in the conversion of the domains into stripes. The cusp is maintained through the entire transition as shown with white arrows. Stripe widths in (D), (H), and (L) are 6.5 ± 0.4 μm, 4.0 ± 0.2 μm, and 4.2 ± 0.2 μm, respectively, similar to those for DPPC:HD mixtures in Fig. 1.

**Fig. 4.**
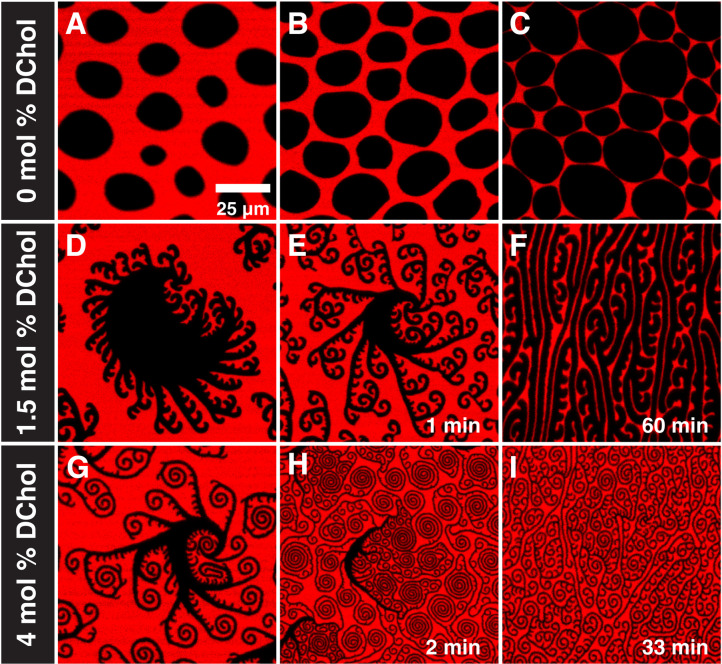
Cholesterol drives the transition. Representative images for 3:1 r-DPPC:HD (**A** to **C**), 9:1 r-DPPC:HD with 0.5 mol % DChol (**D** to **F**), and 9:1 r-DPPC:HD with 4.0 mol % DChol (**G** to **I**). (A to C) Without DChol, r-DPPC:HD domains nucleate below 2 mN/m and retain their semicircular shape with a single cusp throughout coexistence up to a surface pressure of 10 mN/m. No fingering instability is observed nor a transition to stripes. The same characteristic semicircular morphology is observed in films without DChol and in ternary systems containing DChol at surface pressures ≤6 mN/m (compare Figs. 1A and 4A). (D to I) Only with the addition of DChol are finger instabilities and shape transitions observed. Increasing the DChol composition causes a decrease in the onset surface pressure of the finger instabilities [compare (D) and (G)], increases the degree of finger branching and spiraling [compare (E) to (H)], and decreases the equilibrium stripe width [compare (F) and (I)]. DChol is line active and lowers the line tension of the L_C_-L_E_ interface allowing for complex patterns.

Figure 1 shows confocal fluorescence images of the marked changes in L_C_ domain morphology in ternary mixtures of chiral r-dipalmitoylphosphatidylcholine (r-DPPC), 1-HD, and dihydrocholesterol (DChol) as the surface pressure is increased slowly and then held at 7 mN/m. We use dihydrocholesterol as a substitute for cholesterol to minimize oxidation; there is minimal difference in the morphology or phase behavior on this substitution. The contrast in the images is due to the preferential segregation of Texas Red-DHPE fluorescent lipid dye to the continuous, disordered L_E_ phase, which appears red, while the ordered L_C_ domains exclude the dye and appear black (*5*, *38*, *39*). The polydisperse black L_C_ domains that form at surface pressures <7 mN/m have similar asymmetric circular shapes with a single cusp on one end for all DPPC:HD ratios (Fig. 1G, white arrow), with a rounded side opposite the cusp. There is no indication of a preferred domain radius as predicted by Eq. 2. Following compression to 7 mN/m, the L_C_ domains of all three DPPC:HD ratios undergo a fingering instability that usually erupts from the side opposite the cusp (Fig. 1, B, H, and N). The fingering is reminiscent of a Mullins-Sekerka–type instability in 3D crystals and is suggestive of a decrease in the line tension as the surface pressure is increased from 5 to 7 mN/m (*40*, *41*). The fingers have a counterclockwise rotation similar to domains of pure r-DPPC with or without DChol (fig. S1), which is indicative of long-range chiral orientational ordering in the L_C_ domains (*38*). The chiral carbon in native 1,2-dipalmitoyl-*sn-*glycero-3-*sn*-phosphocholine (r-DPPC) induces a counterclockwise orientational ordering in L_C_ domains that persists over tens of micrometers (Figs. 1 to 4). The positional order with the L_C_ domain is short range, on the order of 100 nm or less, so each domain consists of multiple crystallites. Hence, these L_C_ domains might best be described as hexatic phases, rather than true crystalline phases (*42*, *43*).

The chiral center in DPPC is thought to lead to a chiral twist of the molecular tilt orientation within the domains, as well as heterogeneity in the line tension that is manifested in the noncentrosymmetric shapes in Figs. 1A and 2A (*38*, *44*, *45*). Wulff’s theorem (fig. S2) states that the product of boundary length and local line tension should be constant for an equilibrium domain shape (*46*). Hence, in Fig. 1, the chiral domain shapes that end in a cusp are likely due to heterogeneity in the local line tension, perhaps due to different tilt orientations at the domain boundaries (*45*). Line tension variations on different sides of the fingers induced by chirality cause the fingers to curve; the inside part of the curve is shorter than the outside of the curve suggesting that the line tension is higher on the inside, leading to the spiral shapes of the fingers (*18*).

Over the course of an hour, the initially semicircular domains spontaneously change into high–aspect ratio stripes with well-defined and uniform widths that depend on the DPPC:HD ratio (compare Fig. 1, F, L, and R). A single semicircular domain eventually converts into a single stripe of uniform width that roughly aligns with other stripes throughout the monolayer. Thirty minutes to 1 hour are required to progress from Fig. 1B to Fig. 1F at constant surface pressure. The morphology turns into extended stripes of uniform width after an hour.

Each domain in Figs. 1 to 3, regardless of shape, has a single cusp that persists through the circle-to-stripe transition (white arrows in Figs. 1, G to L; 2, G to K; and 3, E to H). The cusps are likely the result of local divergence in the molecular tilt orientational order (*47*–*49*). As in many tilted liquid crystalline materials, the tilt direction changes discontinuously at a topological “virtual boojum” defect (*50*). The virtual boojum is a conceptual defect similar to image charges in electrostatics. The cusp forms near the part of the boundary closest to the boojum defect where the tilt order diverges. The cusp is also an indication of a heterogeneous line tension around the domain boundary. The cusp is located where the line tension reaches a maxima (derivation and fig. S2) (*47*–*49*). This suggests that there is a coupling between the tilt orientation at the domain boundaries and the magnitude of the line tension. The cusp persists throughout the domain shape transitions, confirming that the molecular tilt orientation is preserved. The single cusp also suggests that one circular domain transforms into one linear domain with minimal domain-domain coalescence due to the long-range electrostatic repulsion between domains.

Figure 2 shows that replacing the chiral r-DPPC with racemic DPPC (rac-DPPC) mixtures eliminates the spiral twist in the domain shapes. Domains spread from 3:1 (Fig. 2, A to F), 5:1 (Fig. 2, G to L), or 9:1 (Fig. 2, M to R) molar ratios of rac-DPPC to HD with 1.5 mole percent (mol %) DChol are axially symmetric. However, as in Fig. 1, a single cusp in each domain (arrows) persists through the transition, confirming that the molecules are tilted and maintain their orientational ordering. The domains remain axisymmetric as the surface pressure is increased and fingers begin to form on both sides of the domains (Fig. 3, B, H, and N). Unlike the domains in Fig. 1 that are spiral, the fingers in the racemic mixture domains are relatively straight and point slightly away from the end of the domain with the cusp. The circular domains elongate symmetrically front and back to form extended straight stripes that are uniform in thickness as confirmed by fast Fourier transforms (FFTs) of the images (Fig. 2, F, L, and R). The three to four orders of reflection in the FFTs are indicative of uniformly spaced stripes. For a fixed surface pressure, increasing the ratio of DPPC to HD in the spreading solution decreases the stripe width.

Figure 3 shows that exchanging PA for HD results in identical morphologies and shape transitions. The observed finger instabilities follow a counterclockwise rotation similar to domains in Fig. 1. Each domain also has a single cusp (white arrows in Fig. 1, G to L) consistent with a molecular tilt. Grazing incidence x-ray diffraction (GIXD) shows that PA and HD form nearly identical pseudo-hexagonal tilted packings with DPPC (*7*).

While HD and PA are interchangeable, the small mole fraction of DChol is a primary driver of the transition. DChol is believed to preferentially locate at domain boundaries, lowering the line tension between phases by somehow smoothing out changes in composition, tilt, or molecular order (*4*, *30*, *31*, *51*). Figure 4 (A to C) shows that the domain shapes and sizes of DPPC:HD monolayers without DChol are similar to those shown in Figs. 1 (A, G, and M) and 3 (A, E, and I) at surface pressures below the onset of the fingering instability. However, in the absence of DChol, increasing the surface pressure does not lead to any fingering instability or transition to stripes. Figure 4 (D to F) shows that 0.5 mol % DChol is sufficient to induce the fingering instability and the transition to stripes. This small mole fraction of DChol could not substantially change the dipole density difference between the phases. The marked changes in domain morphology induced by 0.5 mol % DChol are consistent with preferential segregation to the domain boundary, and this “line-actant” behavior lowers the line tension at low mole fractions, similar to how conventional surfactants lower surface tension at 3D interfaces (*4*, *31*, *51*). Increasing the DChol concentration to 4.0 mol % (Fig. 4, G to I) results in smaller domains with thinner fingers and stripes and the amount of branching and spirals increase. Increasing DChol increases the ratio of domain perimeter to domain area, consistent with a decrease in the line tension.

### Molecular origins of the transition

**Fig. 5.**
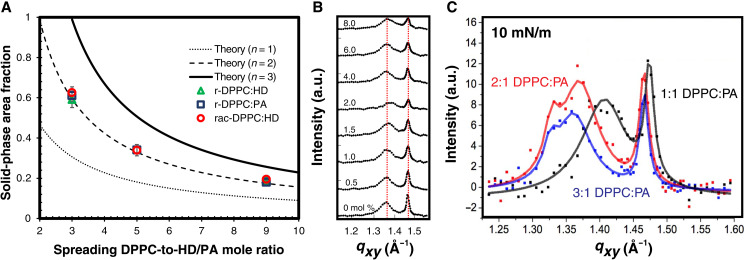
Crystal stoichiometry is independent of the spreading composition. (**A**) The area fraction of the L_C_ phase was calculated for the semicircular domains at 5 mN/m before the onset of the fingering instability (Fig. 1A). Data for 1.5 mol % DChol with r-DPPC and HD, r-DPPC and PA, and rac-DPPC and HD are plotted as green triangles, blue squares, and red circles, respectively. The area fractions are identical and composition insensitive for a given value of *b*, the spreading DPPC–to–HD or PA molar ratio. The lines are plots of Eq. 5 as a function of *b* for various values of *n*, the predicted crystal stoichiometry ratio. Ratios of *n* = 1, 2, or 3 are plotted as fine dash, dash, and solid curves, respectively. The ratio of 2:1 DPPC to HD (or PA) is consistent with the measured L_C_ area fractions. (**B**) GIXD shows that increasing DChol content from 0 to 8 mol % does not change the lattice spacing of DPPC:HD mixtures. HD excludes DChol from the DPPC lattice so the DChol segregates to the L_E_ phase (*57*). a.u., arbitrary units. (**C**) GIXD shows that, in mixtures containing r-DPPC, PA, and DChol, the diffraction pattern is similar for spreading ratios of 2:1 and 3:1 r-DPPC:PA, whereas the 1:1 r-DPPC:PA mixture exhibits a markedly different pattern, supporting the claim that the crystal stoichiometry is 2:1 [redrawn from (*39*)].

**Fig. 6.**
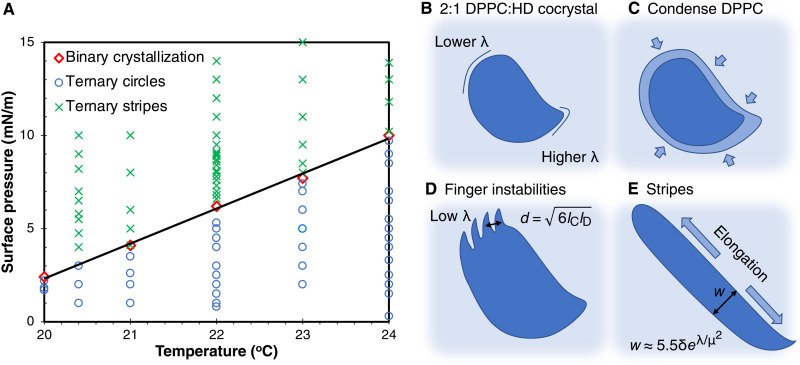
Binary crystallization diagram predicts ternary equilibrium shapes. (**A**) The line shows the onset surface pressure for the L_C_ phase in a mixture of r-DPPC and 1.5 mol % DChol (red diamonds). For π’s above the line, r-DPPC forms L_C_ domains in an L_E_ matrix; for π’s below the line, only L_E_ phase is present. The coexistence line dictates the transition between circles and stripes in ternary mixtures of r-DPPC, HD, and DChol. Compositional changes at the domain boundaries on crossing the coexistence line likely decrease the line tension, which initiates the morphology transition. (**B**) For π < 6 mN/m, domains are asymmetric with a line tension that varies around the boundary; line tension is likely highest near cusp and lowest opposite. The domain composition is 2:1 DPPC:HD, with excess DPPC and all the DChol remaining in the L_E_ phase. (**C**) As the surface pressure is increased above the binary coexistence line [black line in (A)], the boundary of the 2:1 DPPC:HD domains becomes epitaxially enriched with DPPC, which causes a decrease in the line tension. (**D**) The reduced line tension enables a Mullins-Sekerka–type fingering instability. The finger spacing is given by $d\sim \sqrt{l_{C}l_{D}}$ in which *l*_C_ is a capillary length that depends on the line tension and *l*_D_ is a diffusion length that depends on the DPPC diffusivity and the crystal growth velocity (see Fig. 7). (**E**) As the system reaches equilibrium, the fingers anneal away, and the domains elongate into stripes of uniform width with spacing w≈5.5δeλμ2.

The transition between compact semicircular domains at surface pressures less than 7 mN/m and extended stripe domains at greater than 7 mN/m requires a mechanism to decrease the line tension over a narrow range of surface pressure at constant overall monolayer composition. One such mechanism would be the gradual change in the composition of the L_C_ domain boundary over this surface pressure range (Figs. 5 and 6). In DPPC L_C_ phase crystals, the saturated 16-carbon alkane chains pack in a tilted hexagonal lattice; the tilt angle is related to the mismatch in cross-sectional area between the alkane chains and the phosphocholine headgroup (fig. S3) (*7*, *43*, *52*). The 16-carbon alkane chains of HD or PA are identical to those of DPPC and can pack into the DPPC chain lattice, while the relatively small alcohol or fatty acid headgroup can compensate for the phosphocholine headgroup area mismatch, reducing the overall monolayer tilt. This stabilizes the crystal by increasing the favorable van der Waals interactions between the alkane chains (*7*, *39*, *53*–*55*). The increased van der Waals interactions stabilize the L_C_ phase and cause the DPPC:HD or DPPC:PA domains to nucleate at surface pressures ≤1 mN/m, compared to the ~6 mN/m for pure r-DPPC L_C_ phase at similar temperatures (fig. S1).

However, because of the complex, 3D zigzag shape of all-trans alkane chains in the crystal, the molecules cannot pack with an arbitrary tilt to match head and tail areas (fig. S4). Certain discrete tilt angles provide an optimal chain packing to maximize the van der Waals interactions between chains, and several close-packing structures of alkanes are known (*55*, *56*). However, even considering the various discrete tilt angles allowed for all of the classes of packing, not every headgroup can be easily accommodated by a uniform crystalline alkane structure. This can lead to particular stoichiometric ratios of DPPC to HD being preferred to provide the optimal packing to match headgroup and tailgroup areas while accommodating the 3D shapes of the alkane tails (*43*, *54*, *55*).

The domain shapes are quite similar between the images in Fig. 1 (A, G, and M), which also suggests that all the L_C_ crystals may have the same stoichiometry regardless of the composition of the spreading solution. This is consistent with GIXD that shows that DPPC cocrystallizes with PA with the same lattice parameters for 2:1 and 3:1 DPPC:PA spreading solutions (Fig. 5C) (*7*, *39*). GIXD shows that increasing DChol from 0 to 8 mol % in 3:1 r-DPPC:HD monolayers does not change the lattice spacing, suggesting that HD prevents DChol from entering the DPPC:HD/PA cocrystal lattice (Fig. 5B) (*57*). Hence, the DChol resides in the L_E_ phase for these surface pressures.

If the cocrystal of DPPC and HD (or PA) grows at a fixed stoichiometric ratio, then mole balance arguments can determine the cocrystal stoichiometric ratio based on the area fraction of the L_C_ phase as a function of the spreading composition (fig. S5). Figure 5A shows that the L_C_ (black) domain area fraction at 5 mN/m, just before the onset of the fingering transition (Fig. 1, B, H, and N), increases nonlinearly with decreasing DPPC:HD or PA ratios in the spreading solution. The area fraction of black L_C_ phase is identical for r-DPPC:HD, r-DPPC:PA, and rac-DPPC:HD at a given spreading solution ratio.

For *N* molecules of HD and *bN* molecules of DPPC in the spreading solution deposited to form the monolayer, the area fraction of L_C_ phase as a function of *b* for a crystal with *n* ≠ *b* can be calculated, where *b* is the DPPC–to–HD or PA ratio of the spreading solution and *n* is the stoichiometric ratio of DPPC to HD or PA in the L_C_ crystal. Isotherms and x-ray diffraction show the interfacial area per HD molecule, *a_HD_* ~ 0.2 nm^2^ in ordered phases. *a_DC_* ~ 0.45 nm^2^ is the area per DPPC molecule in the L_C_ phase (black), with *a_DL_* ~ 0.75 nm^2^ for DPPC in the L_E_ (red) phase. *a_c_* ~ 0.25 nm^2^ is the area per molecule of DChol. HD expels DChol to the L_E_ phase in mixtures with DPPC (Fig. 5B) (*57*), so all cholesterol is assumed to be in the L_E_ phase. The total image area of L_C_ (black) phase is *A_B_*

$$A_{B}=Na_{\mathrm{HD}}+\mathrm{nN}a_{\mathrm{DC}}$$ (3)

The total image area of the L_E_ (red) phase is *A_R_*, assuming any residual DPPC and all the *j* mol fraction of DChol resides in the LE phase

$$A_{R}=N(b-n)a_{\mathrm{DL}}+\frac{j(b+1)}{\left(1-j\right)}Na_{c}$$ (4)

The area fraction, ϕ, of the L_C_ phase is then

$$\phi =\frac{A_{B}}{A_{B}+A_{R}}=\frac{a_{\mathrm{HD}}+na_{\mathrm{DC}}}{a_{\mathrm{HD}}+na_{\mathrm{DC}}+(b-n)a_{\mathrm{DL}}+\frac{j(b+1)}{\left(1-j\right)}a_{c}}$$ (5)

Figure 5A shows ϕ as a function of *b*, the DPPC:HD ratio in the spreading solution for three different values of the stoichiometric ratio of DPPC:HD in the LC phase crystal, *n =* 1, 2, and 3. The measured values of ϕ fall on the line *n* = 2. The calculated value of ϕ does not change significantly for ±10% variations in *a_HD_*, *a_DC_*, *a_DL_*, or *a_c_*. (Details of the derivation of Eqs. 3 to 5 and fig. S5 showing some of the confocal images used to assess ϕ are in the Supplementary Materials.) This is consistent with GIXD that shows that the diffraction pattern of monolayers spread from 2:1 and 3:1 DPPC:HD ratios is quite similar (Fig. 5C) (*7*, *39*).

### Enriching domain boundaries in DPPC

If the monolayer spread at the interface contains a higher DPPC:HD or DPPC:PA ratio than 2:1 (such as the 3:1, 5:1 and 9:1 DPPC:HD or PA in Figs. 1 to 3), then the 2:1 DPPC:HD crystals (black) nucleate first at near-zero surface pressure, and these domains grow until the limiting HD (or PA) is incorporated into the L_C_ phase domains by 6 mN/m as shown schematically in Fig. 6B. Any excess DPPC remains in the L_E_ phase along with all the DChol and the Texas Red-DHPE dye. The surface pressure at the onset of L_E_-L_C_ coexistence for DPPC or DPPC with DChol is ~6 mN/m, while for DPPC:HD or PA, the onset occurs at <1 mN/m (figs. S1 and S6). The coexistence onset surface pressure does not change significantly for DChol fractions from 0 to 5 mol % (fig. S7). Hence, once the L_E_ phase is depleted of HD or PA by the growing 2:1 DPPC:HD L_C_ phase crystals, increasing the surface pressure to ≥6 mN/m causes the remaining DPPC to condense onto the existing L_C_ domains, thereby either gradually or abruptly changing the composition at the domain boundaries from 2:1 DPPC:HD (or PA) to nearly pure DPPC (Fig. 6C). As no new L_C_ domains were formed in Figs. 1 to 4 on increasing the surface pressure greater than 6 mN/m, DPPC is likely growing epitaxially on the existing DPPC:HD crystals. DPPC and DPPC:HD crystals have the same tilted hexagonal packing of the alkane chains (*7*), with just small differences in tilt, allowing for epitaxial growth. Figure 6A shows that the first fingers form on the 2:1 DPPC:HD L_C_ domains as the system crosses the binary DPPC:DChol coexistence curve as the surface pressure is increased. The compact shapes of the low–surface pressure DPPC:HD L_C_ domains are consistent with a higher line tension than the expanded stripes between the DPPC:DChol L_C_-L_E_ phases (fig. S6). In the ternary domains, the boundary composition is enriched in DPPC from the 2:1 DPPC:HD stoichiometry to nearly pure DPPC as the surface pressure is increased or the temperature is decreased (Fig. 6A) at the binary coexistence surface pressure of DPPC-DChol. This change in composition at the domain boundary lowers the line tension (Fig. 6D), inducing the fingering via the Mullins Sekerka instability. Monolayers spread from 2:1 DPPC:HD with 1.5 mol % DChol do not undergo the circle-to-stripe morphology change nor the fingering instability (fig. S8). The boundary composition would not change as HD would no longer be limiting, and there would be no excess DPPC in the melt.

### Fingering instability due to decrease in line tension

**Fig. 7.**
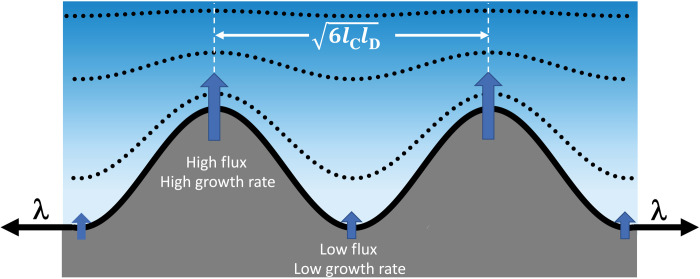
Schematic diagram of the Mullins-Sekerka instability. During L_C_ domain growth (dark gray), concentration gradients are set up in the multicomponent L_E_ phase (blue) as DPPC is depleted near the domain boundaries. A flat growth front is unstable to a sinusoidal fluctuation that compresses the concentration gradient at the tips of the fingers (dotted lines) and expands the gradient in the troughs between fingers. The higher flux at the fingertips increases the growth rate, while the lower flux in the troughs between fingers slows the growth rate, which feeds the instability. Opposing the growth instability is the line tension, λ, which acts to flatten the fingers to minimize the length of the boundary. The instability is favored by low line tensions that arise as the domain boundaries are enriched in DPPC. The separation between fingers is set by $d\sim \sqrt{6l_{C}l_{D}}$ in which lD=Dv is the diffusion length and *l*_C_ = λ/α*_L_*(∆Γ)^2^ is the capillary length, or the ratio of λ to α*_L_*(∆Γ)^2^, where ∆Γ = Γ_LC_ − Γ_LE_ is the difference in concentration between the 2D crystal and the melt and α*_L_* ≡ (∂μ/∂Γ)_Γ = Γ_L__ is the convexity of the free energy near the equilibrium melt concentration, Γ_L_.

The initial growth of regularly spaced “fingers” in Figs. 1 to 4 is due to a version of the classical Mullins-Sekerka growth instability that is responsible for the shapes of snowflakes and dendrites in metal crystals and is mathematically similar to the viscous fingering that occurs in the displacement of high-viscosity fluids by low-viscosity fluids (*40*, *41*). During the growth of the L_C_ domains, concentration gradients are set up in the multicomponent L_E_ phase as DPPC is being depleted near the domain boundaries as it is incorporated into the growing domain. Simultaneously, DChol and the Texas Red dye are being concentrated in the L_E_ phase as these components are excluded from the L_C_ domains. As sketched in Fig. 7, a linear growth front is unstable to a sinusoidal perturbation that compresses the concentration gradient at the tips of the fingers and expands the gradient in the troughs between fingers. In other words, the tips of the fingers extend into higher DPPC concentrations, while the troughs between fingers are in lower DPPC concentrations. As a result, the flux of DPPC onto the growing domain is highest near the fingertips, causing the fingertips to grow faster than the troughs (*40*, *41*), and the instability is enhanced.

Opposing the growth of the sinusoidal perturbation is the line tension that acts to minimize the domain perimeter and keep the boundary flat. Analysis (see the Supplementary Materials) shows that the critical wave number for growth of the fingers increases with decreasing line tension

$$k_{0}=\sqrt{\frac{v\alpha_{L}{\left(\Delta \Gamma \right)}^{2}}{2D_{L}\lambda }}=\frac{1}{\sqrt{2l_{C}l_{D}}}$$ (6)

lD=DLv is the diffusion length, or the ratio of the diffusivity, *D_L_*, of DPPC in the melt to *v*, the average crystal growth front velocity. lC=λαL(∆Γ)2 is the capillary length, or the ratio of the line tension, λ, to α*_L_*(∆Γ)^2^, where ∆Γ = Γ_LC_ − Γ_LE_ is the difference in concentration between the 2D crystal and the melt (Γ=1a, where *a* is the area per molecule) and α*_L_* ≡ (∂μ/∂Γ)_Γ = Γ_L__ is the convexity of the free energy at the equilibrium melt concentration (see figs. S9 and S10). As the line tension decreases, the interface becomes unstable to interfacial perturbations and fingers begin to grow. The fastest growing wave number

$$k^{*}=k_{0}/\sqrt{3};d\sim 1/k^{*}=\sqrt{6l_{C}l_{D}}$$ (7)

predicts the spacing, *d* (Fig. 6E), between the fingertips in Figs. 1 to 4. *d* ~ 7 μm for the 3:1 and *d* ~ 5 μm for the 5:1 and 9:1 spreading solutions from Fig. 1. Increasing the domain growth velocity or decreasing the line tension causes the fingers to be closer together.

As the domain boundaries become enriched in DPPC while the limiting HD is depleted in the melt, the line tension at the domain boundary decreases, causing *k*_0_ and k^*^ to increase and *d* to decrease, leading to the destabilization of the interface and growth of the fingers. The fingers will grow first on the domain boundary with the lowest line tension, which is the curved parts of the domains opposite the cusps (fig. S2). Fingers do not form on the domains at low surface pressure as the line tension between the 2:1 DPPC:HD domains and the melt is too large, making *d* greater than the domain size. DChol is required to induce both the fingers and the circle-to-stripe transition and does so even at 0.5 mol % DChol concentration. DChol likely segregates to the domain boundaries and acts as a line-actant lowering the line tension at the DPPC L_C_-L_E_ boundary (*4*, *51*). The fingers are a dynamic consequence of domain growth; as the supersaturation of the melt is dissipated and the system reaches equilibrium, the fingers stop growing and eventually anneal away to minimize the domain perimeter. However, this analysis does not include the effects of the dipole-dipole repulsion, μ^2^,which may stabilize the high–aspect ratio fingers. McConnell and Moy (*5*, *18*) have shown that the presence of a dipole-dipole repulsion lowers the effective line tension, which would promote the fingering instability as well as the circle-to-stripe transition. An alternate theory describing diffusive growth of a cylindrical crystal replaces the diffusion length, lD=DLv, by *R*, the cylinder radius in Eqs. 6 and 7 (*58*).

### Uniform width stripes—An equilibrium morphology

With time (30 min to several hours), the domains evolve into stripes of uniform width and the fingers anneal away, especially in the rac-DPPC mixtures (Fig. 2, E, K, and Q). Determining the electrostatics for the complex domain shapes in Figs. 1 to 4 is difficult, so, as an approximation, we use a simplified model due to Moy and McConnell (*18*) that describes the transition between a square (line tension dominated) and rectangular (dipole dominated) domain. The initial semicircular domains are compact and have an aspect ratio near one, like the squares, while the final stripes have a large aspect ratio, with the width << length. The free energy, *F*, due to the line tension and dipole-dipole repulsion in a rectangular domain of length, *l*, and width, *w*, is

$$\begin{array}{c} \frac{F}{2\mu^{2}}=\{\frac{\lambda }{\mu^{2}}+\text{ln}\frac{e^{2}\delta }{A}+\text{ln}[w+{\left(w^{2}+l^{2}\right)}^{1/2}]\}w+\\ \{\frac{\lambda }{\mu^{2}}+\text{ln}\frac{e^{2}\delta }{A}+\text{ln}[l+{\left(w^{2}+l^{2}\right)}^{1/2}]\}l-2{\left(w^{2}+l^{2}\right)}^{1/2} \end{array}$$ (8)

in which *A = lw* is the individual domain area, which is roughly constant during the transition, and, as in Eqs. 1 and 2, *e* is the exponential, 2.718 and δ ~ 1.0 nm is the separation between molecular dipoles. This free energy is minimized, *∂F*/*∂w* = 0 with *l* = *A*/*w* for fixed domain area, *A*

$$\begin{array}{c} -\frac{w^{2}}{2\mu^{2}}\frac{\partial F}{\partial w}=A\text{ln}(\frac{A+\sqrt{A^{2}+w^{4}}}{\mathrm{Aw}})-\\ w^{2}\text{ln}(\frac{w^{2}+\sqrt{A^{2}+w^{4}}}{\mathrm{Aw}})+(A-w^{2})\text{ln}(\delta e^{1+\lambda /\mu^{2}})=0 \end{array}$$ (9)

**Fig. 8.**
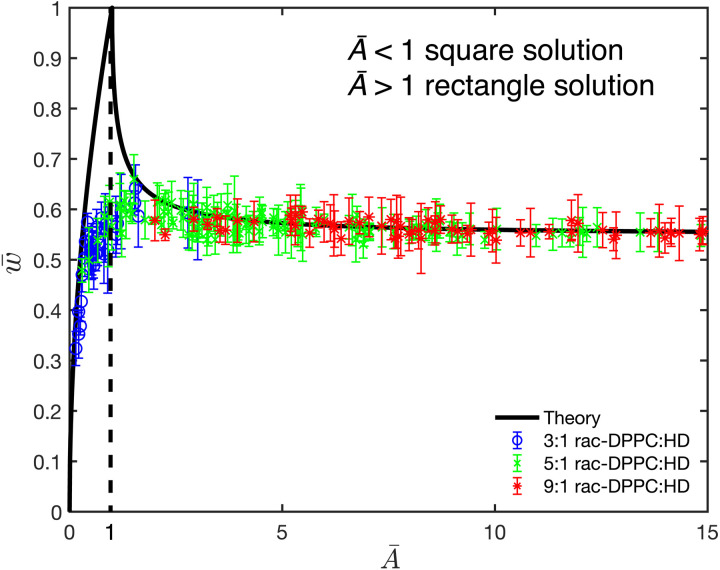
Individual domains reach an equilibrium stripe width. A plot of the dimensionless domain width, $\bar{w}=w/w_{c}$, scaled by the critical width, *w*_c_, versus the scaled domain area, $\bar{A}=A/w_{c}^{2}$, from Eqs. 11 to 13. Here, the shape transition is expected when $\bar{w}=\bar{A}=1$ (dashed line). Theory predicts two solutions: one for square ($\bar{A}<1$) and one for rectangular shapes ($\bar{A}>1$). Ternary mixtures of rac-DPPC and HD at molar ratios of 3:1 (blue circles), 5:1 (green crosses), or 9:1 (red asterisks) with 1.5 mol % DChol show good agreement for $\bar{A}$ values greater than 1 (rectangle shapes). As $\bar{A}$ increases, the rectangular domains approach a uniform width independent of their length that is by set by the competition between λ and μ^2^. For equal–aspect ratio shapes $(\bar{A}<1$), theory and experiment follow the same general trend; however, the transition does not occur at $\bar{w}=1$ because of differences in the energetics of a square (theory) compared to the actual semicircular shape.

Equation 9 has two solution branches (solid lines in Fig. 8): the first being *A* = *w*^2^, corresponding to square domains with *l* = *w*. To determine the second, rectangular branch, Eq. 9 is solved numerically to give the relationship between the domain width and area for *l* ≠ *w*. The two solution branches bifurcate at a critical domain width, *w* = *w*_c_, corresponding to a critical area, $A_{c}\equiv w_{c}^{2}.$ This critical domain width is determined from the second derivative of Eq. 9 for $A_{c}\equiv w_{c}^{2}$

$${\frac{\partial^{2}F}{\partial w^{2}}|}_{w=w_{c},A=w_{c}^{2}}=2\text{ln}(\frac{\delta }{w_{c}}(1+\sqrt{2})e^{\left(\sqrt{2}+\lambda /\mu^{2}\right)})=0$$ (10)

Above this critical width, the square shape becomes higher in energy than the rectangular shape. Solving for *w*_c_ gives

$$w_{c}=\delta (1+\sqrt{2})e^{\left(\sqrt{2}+\lambda /\mu^{2}\right)}\approx 10\delta e^{\lambda /\mu^{2}};A_{c}\approx 100\delta^{2}e^{2\lambda /\mu^{2}}$$ (11)

To illustrate the rest of the rectangular branch, Eq. 9 is scaled by *w*_c_, which defines a dimensionless width and area, $\bar{w}\equiv w/w_{c};\bar{A}\equiv A/w_{c}^{2}$

$$\begin{array}{c} \bar{A}\text{ln}(\frac{\bar{A}+\sqrt{{\bar{A}}^{2}+{\bar{w}}^{4}}}{\bar{A}w})-{\bar{w}}^{2}\text{ln}(\frac{{\bar{w}}^{2}+\sqrt{{\bar{A}}^{2}+{\bar{w}}^{4}}}{\bar{A}\bar{w}})+\\ (\bar{A}-{\bar{w}}^{2})\text{ln}((1+\sqrt{2})e^{\left(\sqrt{2}-1\right)})=0 \end{array}$$ (12)

where we have made use of Eq. 11 to simplify the final term in Eq. 12. The two solution branches are shown in Fig. 8. The square solution to Eq. 12 is $\bar{A}={\bar{w}}^{2}$, and this “square” solution branch in Fig. 8 is given by

$${\bar{w}}_{\text{square}}=\sqrt{\bar{A}};0<\bar{A}\leq 1$$ (13)

The numerical solution for the second or “rectangular” solution to Eq. 12 is also plotted in Fig. 8. The minimum energy solution converts from squares to rectangles at $\bar{w}=\bar{A}=1.$ An asymptotic approximation for the rectangular branch of Eq. 12 that gives good agreement with the numerical solution for $\bar{A}>1.75$is

$$\begin{array}{c} {\bar{w}}_{\text{rectangle}}=0.547+0.114{\bar{A}}^{-1}+0.0563{\bar{A}}^{-2}+\\ 0.0352{\bar{A}}^{-3}+0.0247\;{\bar{A}}^{-4} \end{array}$$ (14)

The leading term is $2e^{\left(1-\sqrt{2}\right)}/(1+\sqrt{2})=0.547,$ which gives the limiting stripe width, *w* = 0.547*w*_c_ ≈ 5.5δ*e*^λ/μ^2^^ for large domain areas or stripe lengths. The domain width is roughly constant for $\bar{A}>5$. As the domain area increases, the stripes lengthen, and Eq. 14 shows that the energy no longer depends on the length of the domains but only on the width of the domains. We see from Figs. 1 to 4 that the final stripe widths are uniform and independent of the stripe length and hence domain area, consistent with Eq. 14 and Fig. 8. Image analysis can provide the area and width of stripe domains for different spreading solutions and surface pressures to determine *w*_c_ from Eq. 14, and then $\lambda /\mu^{2}=N_{\text{Bo}}^{-1}$for the various stripes.

At surface pressures below the transition (Fig. 6B), the semicircular domains shown in Figs. 1 to 4 are such that *A*_domains_ < *A*_c_ ≈ 100δ^2^*e*^2λ/μ^2^^ as the line tension between the 2:1 DPPC:HD L_C_ phase and the DPPC/DChol L_E_ phase is high, making λ/μ^2^ large. The epitaxial growth of DPPC on the original 2:1 DPPC:HD domains decrease the line tension, λ, causing *A*_c_ to decrease and make *A*_domains_ > *A*_c_. These domains transition from the square solution branch (Fig. 8) by the slow conversion of individual square domains into stripes of uniform width, *w* ≈ 0.547*w*_c_. The data for all mixtures show that the striped domains widths versus areas lie along the rectangular branch of the solution to Eq. 14 for $\bar{A}>2$ for all compositions tested. As the domains widths decrease, $\bar{A}$ increases and the domain width approaches *w* ≈ 0.574*w*_c_ ≈ 5.5δ*e*^λ/μ^2^^.

For $\bar{A}<2$, the data do not agree with the predicted bifurcation of the square to stripe solution occurring at $\bar{A}=1$ in Fig. 8. This is not unexpected as the actual shape of the domains is not square in Figs. 1 to 4 but semicircular. Equation 2 describes the energy of an isolated circular domain, which predicts a single optimal radius and domain area for a given value of λ/μ^2^. Unlike the rectangular domains in which both $\bar{w}$and $\bar{A}$ can vary, for circular domains, the continuum theory predicts a single value for the optimal radius and domain area. However, the data for $\bar{A}<2$ do not have any clear optimal value, consistent with previous observations that semicircular and circular domain sizes are polydisperse and likely set by kinetic effects of nucleation and growth.

**Fig. 9.**
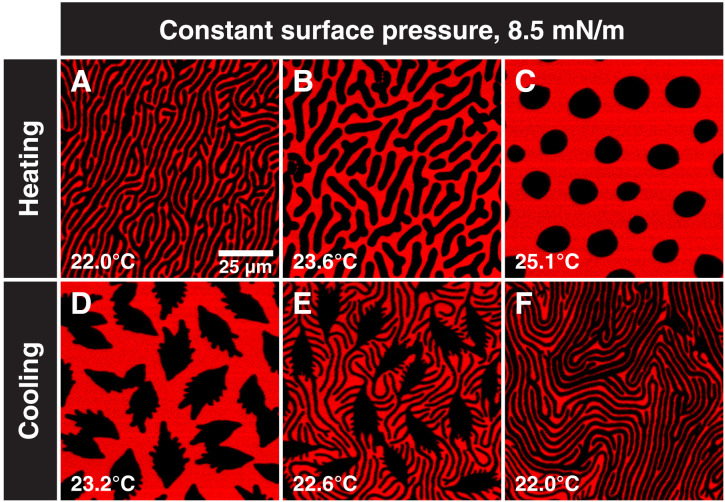
Reversible stripe-to-circle-to-stripe transition by temperature cycling. (**A**) Stripes are the equilibrium configuration at 22°C and a surface pressure of 8.5 mN/m. (**B**) Increasing the temperature to >25°C at 8.5 mN/m places the system above the binary coexistence line in Fig. 6A, causing the stripes to shorten and (**C**) eventually revert to the same semicircular shapes present at low surface pressures in Figs. 1 to 4. This suggests that the DPPC layer that grew on the 2:1 DPPC:HD domains melted away, leading to a higher–line tension boundary. (**D**) The cooling process involves growth of the low–line tension DPPC boundary, which, in turn, initiates the Mullins-Sekerka fingering instability. (**E**) As the system cools, the fingers grow similar to when the surface pressure is increased at a constant temperature. (**F**) Cooling the monolayer back to 22°C causes the stripes to re-form with similar width to (A). The process can also be reversed by changes in surface pressure at constant temperature (fig. S11).

The results presented in Fig. 8 are consistent with the stripe domains being an equilibrium shape with a minimum energy configuration dictated by the ratio of line tension to dipole density difference, $\lambda /\mu^{2}=N_{\text{Bo}}^{-1}$. True equilibrium requires that the final morphology be path independent; we find identical stripe widths if we cross the binary coexistence curve by increasing the surface pressure at constant temperature or by lowering the temperature at constant surface pressure (Fig. 6A). Figure 9 shows the first temperature cycle at a constant surface pressure of 8.5 mN/m for a spreading solution of 9:1 rac-DPPC:HD + 1.5 mol % DChol that followed a constant pressure cycle to reach the stripe morphology. Figure 9A shows stripes of uniform thickness at 22.0°C and 8.5 mN/m. If we raise the temperature while keeping the surface pressure constant at 8.5 mN/m, the stripes shorten and thicken (Fig. 9B), until at 25.1°C, and the semicircular domains with single cusps are recovered (Fig. 9C). Reducing the temperature back to 22°C induces the fingering instability and the eventual conversion back to stripes (Fig. 9F) that are very similar in width as Fig. 9A. The stripe widths are independent of the pathway taken, consistent with an equilibrium morphology. The transition also shows that a single stripe forms from a single circular domain and that the cusp defect is retained throughout the cyclic path. The fingering instability in Fig. 9 (D and E) only occurs on cooling while the DPPC is being condensed onto the crystalline domains, which confirms that these are growth instabilities induced by the decreasing line tension. This cyclic return to the same structures is quite different from those previously found in most L_C_-L_E_ or L_D_-L_O_ systems that form circular domains, in which the size distribution of the domains is determined by the nucleation and growth kinetics rather than the minimum energy radius from Eq. 2 (*14*). Figure S11 shows that a similar stripe-to-circle morphology change can be obtained by decreasing the surface pressure at a constant temperature consistent with Fig. 6A.

**Fig. 10.**
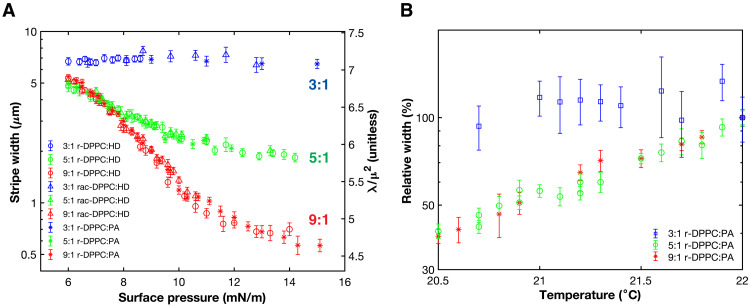
Surface pressure and temperature dependence of the stripe width. (**A**) Stripe domains take on an equilibrium shape dictated by a minimum energy configuration set by the ratio of line tension to dipole density difference squared, λ/μ^2^. From the stripe widths, *w*, an estimate of λ/μ^2^ can be obtained from $\text{ln}\frac{w}{5.5\delta }\approx \lambda /\mu^{2}=N_{\text{Bo}}^{-1}$ for δ = 1 nm. At all three spreading ratios of DPPC to HD or PA, *b* = 3 (blue points), 5 (green points), and 9 (red points) containing 1.5 mol % DChol, λ/μ^2^ was indistinguishable when PA replaced HD or rac-DPPC replaced r-DPPC. The initial value of λ/μ^2^ is independent of composition; however, this ratio does depend on *b* as the surface pressure is varied. For *b* = 3, λ/μ^2^ is independent of surface pressure, whereas, for *b* = 5 and *b* = 9, λ/μ^2^ decreases before reaching a plateau, with *b* = 9 reaching the lowest values. The line width likely depends on the composition at the L_E_-L_C_ boundary, which changes as a function of surface pressure. (**B**) Domain stripe widths are normalized relative to stripe width at 22°C. All compositions include 1.5 mol % DChol. Mixtures containing 3:1 r-DPPC:PA (blue squares) show minimal temperature dependence, while both 5:1 r-DPPC:PA (green circles) and 9:1 r-DPPC:PA (red asterisks) show an almost identical temperature dependence over the presented temperature range.

Figure 10 shows the surface pressure and temperature dependence of the stripe width plotted in terms of the Bond number $\text{ln}\frac{w}{5.5\delta }\approx \lambda /\mu^{2}=N_{\text{Bo}}^{-1}$. For these calculations, the minimum distance between dipoles, δ = 1 nm. At all three ratios of DPPC to HD, DPPC to PA, and rac-DPPC to HD, $N_{\text{Bo}}^{-1}$ and the stripe width were indistinguishable when PA replaced HD or rac-DPPC replaced r-DPPC over the entire range of surface pressure. This suggests that neither the composition of the binary crystals that initially formed the domains at low surface pressures nor the chirality of the domains determine the stripe width. However, the stripe width does depend on the ratio of DPPC to HD or PA in the spreading solution. At the transition surface pressure of 6 mN/m, the three systems have roughly the same $N_{\text{Bo}}^{-1}$ of ~6 to 7, suggesting that this $N_{\text{Bo}}^{-1}$ marks the boundary between circles and stripes. For the 3:1 DPPC:HD, the stripe width of 7 ± 1 μm and $N_{\text{Bo}}^{-1}$ ~ 7 were independent of surface pressure. For the 9:1 DPPC:HD ratios, the stripe width decreased logarithmically from 5.5 μm to the optical resolution limit of <0.5 μm with surface pressure and hence roughly linearly with λ/μ^2^ until leveling off for surface pressures greater than 12 mN/m. The 5:1 DPPC:HD ratio also decreased logarithmically from 5 to 2 μm with surface pressure and, hence, also linearly with λ/μ^2^ to surface pressures of ~10 mN/m before leveling off. This may have to do with the gradually increasing DChol fraction in the L_E_ melt as more DPPC is added to the growing L_C_ domains.

Figure 10B shows that the stripe width is temperature sensitive for the 9:1 and 5:1 at constant surface pressure. The plot shows the stripe width normalized by the stripe width for that DPPC:PA ratio at 22°C. Decreasing the temperature does not change the stripe width of the 3:1 DPPC:PA mixture. The 5:1 and 9:1 DPPC:PA decrease significantly with decreasing temperature. It is difficult to measure the composition of the L_C_ domain and L_E_ melt, so we have not made a direct correlation between local compositions and Bond number. However, as DPPC is depleted from the L_E_ phase, the fraction of DChol and Texas Red-DHPE increase, which may be the origin of the surface pressure and temperature dependence of the stripe width. At high enough DChol mole fractions, DPPC-DChol monolayers convert into disordered liquid L_d_ phases, which may act to lower the line tension between the L_C_ phase and the melt.

## DISCUSSION

We show here that monolayers spread from mixtures of DPPC, HD (or PA), and DChol are among the few systems that spontaneously form domain morphologies that quantitatively agree with the predictions of continuum theory based on the competition between the energetic contributions of dipole-dipole interactions and line tension (*5*). The stripe morphology and equilibrium stripe width are the same regardless of how the stripes are approached, either by changes in surface pressure or changes in temperature.

Our results are consistent with the hypothesis that the transition between circles and stripes is due to the change in line tension caused by the growth of a DPPC-rich phase at the domain boundaries. HD and PA, which have small headgroups but the same 16-carbon saturated alkane chains as the large headgroup DPPC, cocrystallize with DPPC at a 2:1 DPPC:HD stoichiometric ratio, which reduces the tilt of the crystal lattice imposed by the difference in headgroup and chain packing area requirements. Monolayers spread from solutions with DPPC in excess of the 2:1 DPPC:HD stoichiometric ratio first nucleate L_C_ domains with 2:1 DPPC:HD ratios that have a high line tension with their associated L_E_ phase. Eventually, the limiting HD is depleted from the L_E_ phase and the L_C_ domain composition at the boundary changes from the 2:1 DPPC:HD stoichiometry to more nearly pure DPPC. In the presence of as little as 0.5 mol % dihydrocholesterol, this decreases the line tension at the boundary sufficiently to induce a Mullins-Sekerka–type growth instability that results in fingers of well-defined spacing to erupt from specific regions of the domain boundary with the lowest line tension. The fingers are likely stabilized by the dipole density difference that prefers high–aspect ratio shapes.

The domain growth ends as the system reaches equilibrium at constant surface pressure and temperature, causing the fingers to anneal away to leave behind extended rectangular stripes consistent with minimum energy solutions based on the competition between line tension and the dipole density difference described by Eqs. 8 to 14. For sufficiently large domains, the energy depends only on the domain width and is asymptotically independent of the domain length. This equilibrium width provides estimates of the Bond number, *N*_Bo_ = μ^2^/λ for the L_C_-L_E_ phase that depends on composition, surface pressure, and temperature. A single cusp singularity is present in each domain throughout the transition consistent with long-range tilt orientational order that leads to a heterogeneous line tension that is a maximum at the cusp and a minimum on the side opposite the cusp. These cusps are similar to those described as virtual boojums in the liquid crystal literature (*50*). Exchanging rac-DPPC for native r-DPPC eliminates the chiral twist of the domains, leaving behind stripes that are straight for hundreds of micrometers. This circle-to-stripe equilibrium transition can equilibrate relatively easily as each stripe domain achieves its optimal width by relatively fast self-reorganization rather than the much slower global Ostwald ripening required of circular domains. As the domain energy is asymptotically independent of the stripe length, each domain can reach an optimal width without needing to exchange material with other domains.

Future work will show if these stripes, which have distinctly different ends determined by the tilt orientation, can be ordered in flow or electric fields and could act as templates for organizing membrane proteins or other molecules with different solubility in L_C_ versus L_E_ phases. This may provide an interesting model system for quantitative studies on the role of monolayer organization on the reaction-diffusion of proteins in monolayers and bilayers. This work also shows the dominant effect of cholesterol in regulating domain morphology even at trace concentrations. These alterations in morphology can explain the effects that cholesterol has on the interfacial shear and dilatational rheology of LS films and raise further questions on the role of cholesterol in replacement LSs (*8*, *39*, *52*).

## MATERIALS AND METHODS

### Experimental design

This study used confocal fluorescence microscopy to analyze domain morphology in phase-separated lipid-cholesterol films at the air-water interface in a Langmuir trough.

### Materials

Ultrapure water (resistivity ≥18.2 megohm·cm) from a Milli-Q Direct-Q 3 UV-R system (MilliPore) was used as the subphase for all experiments. 1,2-Dipalmitoyl-*sn*-glycero-3-phosphocholine (r-DPPC, R-enantiomer) (Avanti Polar Lipids, Alabaster, AL) or 1,2-dipalmitoyl-rac-glycero-3-phosphocholine (rac-DPPC) (MilliporeSigma, Germany) was mixed with PA (MilliporeSigma, Germany) or 1-HD (MilliporeSigma, Germany) in various molar ratios with small mole fractions of dihydrocholesterol (DChol) (MilliporeSigma, Germany) in chloroform solution. Dihydrocholesterol was used instead of cholesterol to minimize oxidation but has little impact on monolayer morphology or phase behavior ((*5*)). Fluorescence contrast was achieved via the addition of 0.75 mol % Texas Red DHPE [*N*-(Texas Red sulfonyl)-1,2-dihexadecanoyl-*sn*-glycero-3-phosphoethanolamine] (Invitrogen, Grand Island, NY). The Texas Red-DHPE is excluded from L_C_ domains and segregates to L_E_ domains, providing image contrast. Replacing the insoluble Texas Red DHPE dye with soluble Rhodamine 123 in the subphase does not change the fingering instabilities or the circle-to-stripe transition (fig. S12) (*59*).

### Langmuir trough

A custom-built Langmuir trough allows for simultaneous surface balance measurements and confocal microscopy visualization of monolayer domain morphology. The trough is made from milled polytetrafluoroethylene (PTFE). A continuous, stainless steel ribbon barrier is used to change the monolayer area and to prevent material leakage. The maximum and minimum trough areas are 145 and 45 cm^2^, respectively. The surface pressure (reduction in surface tension from a clean air-water interface) was recorded with a filter paper Wilhelmy plate tensiometer (Riegler and Kirstein, Germany) and calibrated using ultrapure water at 22°C. An infrared thermocouple (OS36SM, Omega Engineering) measured the interfacial temperature. A circulating water bath (Thermo Fisher Scientific) provided rough temperature control, and a flexible resistive heater (Minco) underneath the PTFE trough provided fine temperature control. A custom-milled Delrin cover was used to limit evaporation and reduce interfacial motion due to external air currents in the room.

### Isotherms

Mixtures of DPPC, HD (or PA), DChol, and Texas Red DHPE were diluted to a total concentration of 1 mg/ml in HPLC Plus grade chloroform (MilliporeSigma, Germany) to form a spreading solution. The spreading solution was deposited dropwise from a Hamilton syringe (Reno, Nevada) onto the air-water interface of the Langmuir trough described above. One hour was allowed for solvent evaporation before film compression. The trough was compressed at a constant speed of 10 cm^2^/min for all experiments. A computer interface written in LabVIEW (National Instruments, Austin, TX) handled trough barrier control, temperature control, and data collection.

### Confocal fluorescence microscopy

Laser scanning confocal microscopy was performed using a C1 confocal scan head fitted to an Eclipse 80i upright microscope (Nikon Instruments). A Nikon plan apochromatic 20× objective (1.0-mm working distance, 0.75 numerical aperture, air immersion, 170-μm coverslip correction) was used for all experiments. The objective was heated using a flexible, resistive heater to prevent condensation onto the objective for temperature-dependent experiments.

### Statistical analysis

All image analysis was performed using ImageJ. The reported error in all presented analyses is the SD calculated from a minimum of 30 images from at least three different experiments. Domain shape analysis was performed on confocal images that were converted to 8-bit and rendered binary using the Huang thresholding method. The area fraction of L_C_ phase was determined using the built-in ImageJ “Area fraction” function. Domain area and perimeter were determined by ImageJ built-in functions. The domain stripe widths were determined via manual measurements (15 measurements per stripe, 100 stripes per surface pressure or temperature condition) and corroborated with image analysis (see the Supplementary Materials for details).

## Notes

View/request a protocol for this paper from *Bio-protocol*.
