# Supplementary material for: Spontaneous evolution of equilibrium morphology in phospholipid-cholesterol monolayers
Source: Sci Adv. 2022 Apr 6;8(14):eabl9152. doi: 10.1126/sciadv.abl9152 (PMC8986108; doi:10.1126/sciadv.abl9152)
Supplement: Supplementary file 1 — Supplementary Text Figs. S1 to S12 [file sciadv.abl9152_sm.pdf]

Science Advances
AAAS

Supplementary Materials for

Spontaneous evolution of equilibrium morphology in phospholipid-cholesterol monolayers

Cain Valtierrez-Gaytan, Joseph M. Barakat, Mitchell Kohler, Khanh Kieu, Benjamin L. Stottrup, Joseph A. Zasadzinski*

*Corresponding author. Email: zasad008@umn.edu

Published 6 April 2022, *Sci. Adv.* **8**, eabl9152 (2022)
DOI: 10.1126/sciadv.abl9152

This PDF file includes:

Supplementary Text
Figs. S1 to S12

Supplementary Text

Morphology of r-DPPC and Binary Mixtures

Figure S1 A to D shows the evolution of the L_C domains (black) of pure r-dipalmitoylphosphatidylcholine (r-DPPC) in a continuous red L_E phase. The LE-LC coexistence surface pressure depends on temperature; at 22° C, this surface pressure ranges from 7 – 11 mN/m. To visualize the phase separation, a small mole fraction of Texas Red – DHPE is added to the chloroform spreading solution. The Texas Red-DHPE segregates almost exclusively into the fluid

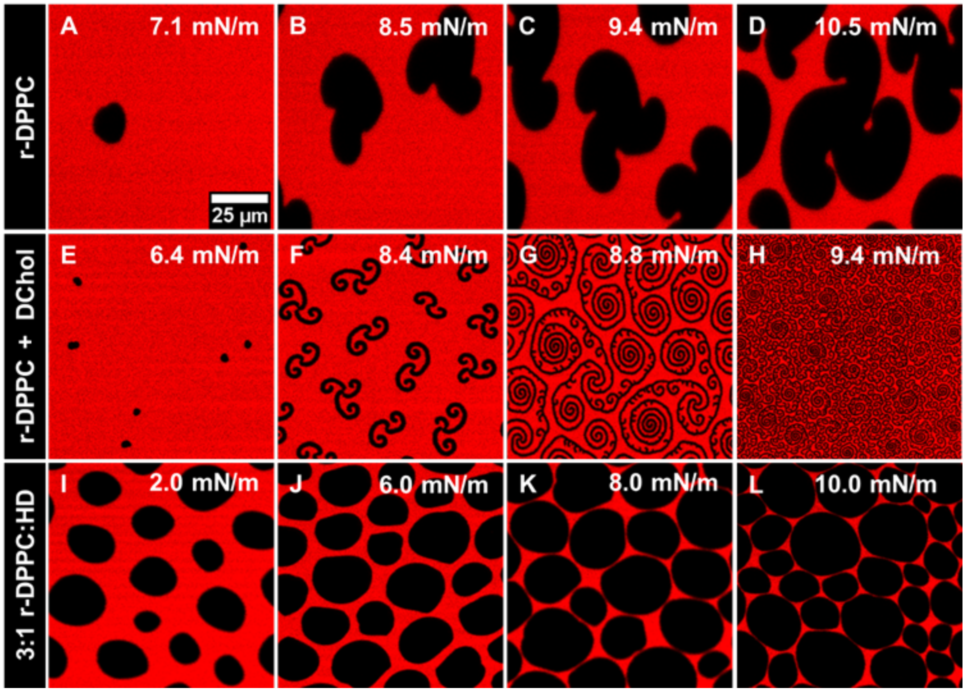

Figure S1. r-DPPC monolayers and binary mixtures of DPPC with dihydrocholesterol or 1-hexadecanol. Confocal microscopy images of phase separated films; black: semi-crystalline domains of liquid condensed (L_C), red: liquid expanded (L_E) matrix. The preferential partitioning of 0.75 mol % Texas Red DHPE dye into the LE phase provides the contrast between phases. (A to D) Images for dipalmitoylphosphatidylcholine (r-DPPC) (at 23 °C). **A**) Domain nucleation begins at a surface pressure of 7 ± 0.2 mN/m at **23** °C (6 ± 0.2 mN/m @ 22 °C) and **B**) further compression leads to multi-lobe branching that (**C** and **D**) ultimately results in characteristic chiral 3-lobe triskelion structures that grow larger with increasing surface pressure. (E to H) The addition of 1.5 mol % dihydrocholesterol (DChol) results (**E** and **F**) in smaller domains with similar multi-lobe structures. **G**) As the film is compressed, the line width at a given surface pressure significantly decreases, and the degree of branching increases. **H**) At high surface pressures, the line width decreases below our optical resolution. (**I** to **L**) The addition of 1-hexadecanol (HD) (3 to 1 r-DPPC to HD molar ratio) results in domain nucleation at near-zero surface pressures consistent with a more stable L_C crystal. The domains are semi-circular and have a singular cusp that is maintained as the domain grows with increasing surface pressure. All images are 100 × 100 µm.

L_E phase and is expelled from the semi-crystalline L_C phase. r-DPPC domains form a characteristic triskelion pattern with counter-clockwise rotation. This rotation is due to the chiral center of the DPPC molecule that induces a twist in the tilt axis orientation. The positional order of the DPPC is short range, on the order of 100 nm or less, so each domain consists of multiple crystallites. However, Figs. S1A-D show that the orientational order that leads to the characteristic counter-clockwise orientation persists for tens of microns. Hence, DPPC and other L_C phases might best be described as hexatic phases, rather than true crystalline phases (*42, 43, 52*).

When 1.5 mol % dihydrocholesterol (DChol) is added to DPPC (Fig. S1 E-H), the coexistence surface pressure is similar, but the domains arms thin (compare B and C to F and G) and form tight spiral loops, twisting mainly in the counter-clockwise direction. This change from the compact r-DPPC domains to the more extended stripes is indicative that even small amounts of DChol dramatically decrease the line tension at the domain boundaries. At higher surface tensions, the stripes can no longer be resolved with the confocal microscope.

In mixtures of r-DPPC with 1-hexadecanol (Fig. S1 I-L), the extended arms of the pure r-DPPC disappear in favor of a more compact asymmetric circular domain. The domains typically end in a cusp at one end with the opposite end rounded. This more compact shape suggests that the DPPC:HD domains have a higher line tension than the pure r-DPPC domains as the perimeter to area ratio decreases. This domain shape is retained to higher surface pressures (I-L). The asymmetric shape and cusp are indicative of a line tension that varies around the domain perimeters as shown in the following sections.

Wulff's Theorem and the Cusp Singularity

Wulff's theorem states that the energy of a crystalline boundary is proportional to its distance from the center of the crystal. Burton, Cabrera, and Frank (Appendix D of Ref. (*46*)) showed that this distance, $r(\theta)$, can be related to a spatially varying, heterogeneous line tension, $\lambda(\theta)$, by

$$(k_B T \ln \alpha)[r''(\theta) + r(\theta)] = \lambda''(\theta) + \lambda(\theta) \qquad (S1)$$

for which the solution is

$$r(\theta) = \frac{\lambda(\theta)}{k_B T \ln \alpha} + C \sin(\theta - \psi) \qquad (S2)$$

where θ is the polar angle around the perimeter and α, ψ, and C are constants to be set by the particular boundary conditions. The x,y positions of the domain are given by the parametric curves:

$$x(\theta) = r(\theta)\cos\theta - r'(\theta)\sin\theta \qquad (S3)$$

$$y(\theta) = r(\theta)\sin\theta + r'(\theta)\cos\theta \qquad (S4)$$

Rudnick and Bruinsma (*49*) considered the model line tension (Figure S2A),

$$\lambda(\theta) = \lambda_0 e^{\beta\cos\theta} \qquad (S5)$$

for which the associated shape (setting the arbitrary constant $C = 0$) is

$$x(\theta) = r_0(\cos\theta + \beta\sin^2\theta)e^{\beta\cos\theta} \qquad (S6)$$

$$y(\theta) = r_0(1 - \beta\cos\theta)\sin\theta\, e^{\beta\cos\theta} \qquad (S7)$$

where $r_0 \equiv \lambda_0/k_B T \ln \alpha$. As β increases, the line tension becomes more strongly peaked at $\theta = 0$. At the critical point $\beta = 1$, the function $r''(\theta) + r(\theta)$ undergoes a change in sign at $\theta = 0$, indicative of the onset of a cusp (Figure S2B).

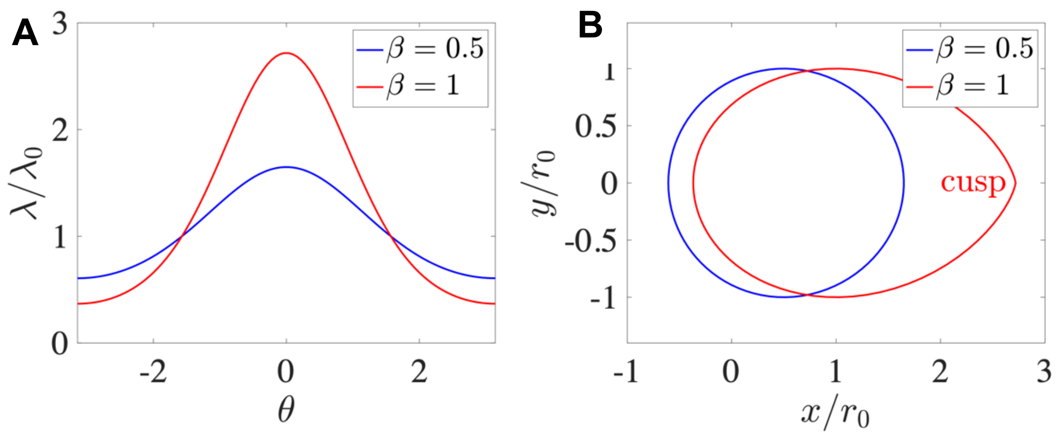

Figure S2. Variation in line tension produces cusps. A) Model variable line tension from Eqn. S5. The line tension is a maximum for θ = 0 which depends on the value of β, and decreases symmetrically with ± θ. **B**) Calculated domain shapes (Eqns. S6, S7), with the cusp emerging (for $\boldsymbol{\beta \geq 1}$) at the pole where the line tension is peaked, which corresponds to θ = 0. These calculated domains are similar to those in Fig. 2 for rac-DPPC. The chirality of the r-DPPC makes the domain shapes more complex and breaks the axial symmetry.

The variation in line tension and the resulting cusps may result from the tilt orientation at the domain boundary, with a singularity in the tilt orientation near the cusp. If the tilt of the molecules prefers to be oriented in a particular direction with respect to the domain boundary throughout the domain, the tilt orientation at the cusp must re-orient quickly and effectively forms an orientational defect known as a "virtual boojum". This virtual boojum is equivalent to a +1 disclination defect conceptually located just outside the domain boundary near the cusp (*49, 50*). The tilt orientation throughout the domain can be thought of as originating from the virtual boojum.

Molecular geometry and the preferred DPPC:HD ratio

Figure S3 shows a schematic of the origins of tilt in the DPPC-HD (or DPPC-PA) lattice. For simple, unstructured molecules as in Fig. S3, if the projected area of the alkane chains (**a** in Fig. S3A) is smaller than the projected area of the head group (**b** in Fig. S3A), gaps would exist between the alkane chains (red arrows). Tilting the molecules relative to the interface can lead to closer packing, thereby increasing the van der Waals attractive interactions and stabilizing the crystal (Fig. S5B) (*7, 54, 55*). GIXD results show that the average molecular tilt in DPPC in the L_C phase is ~ 25° (*7*); from Fig. S3B, $\cos 25° = {}^{a}/_{b} = 0.9$. The sixteen carbon alkane chains of HD or PA are identical to those of DPPC and can pack into the DPPC chain lattice, while the alcohol or fatty acid

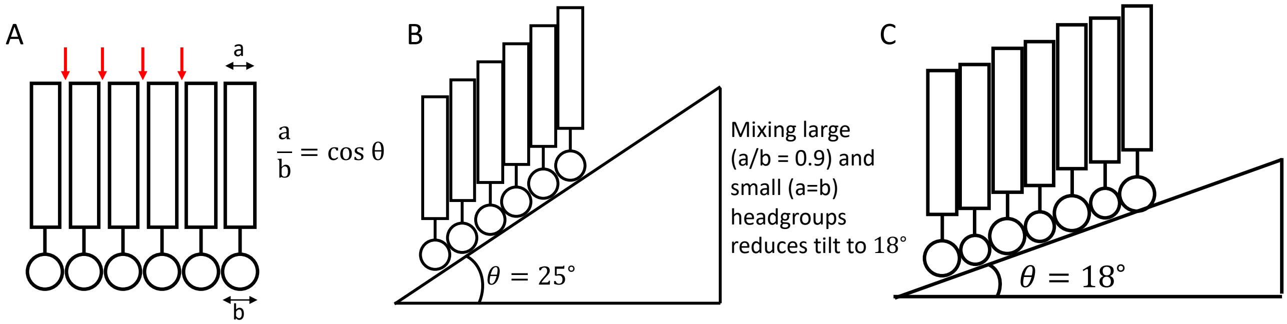

Figure S3. Simple models for molecular tilt. A) Schematic diagram of simple, unstructured lipid molecules with different tailgroup (**a**) and headgroup (**b**) projected areas, here ${}^{a}/_{b} = 0.9$. For vertical packing, a gap would exist (red arrows) between the molecules and minimal van der Waals interactions. **B)** If the molecules are tilted at ${}^{a}/_{b} = \cos\theta = 0.9$, close packing occurs for $\theta = 25°$. **C)** Mixing large and small headgroup molecules together reduces the overall tilt, increasing the van der Waals interactions as there is less offset between the molecules. For more realistic molecules with 3-dimensional structure, arbitrary tilt angles are not compatible with closest packing (55).

headgroups are relatively small so $a \approx b$ which partially compensates for the phosphocholine headgroup area mismatch, reducing the overall monolayer tilt (Fig. S3C). This stabilizes the crystal by increasing the favorable van der Waals interactions between the alkane chains as there is more chain overlap for decreasing tilt (Fig. S3C). This causes the DPPC:HD or DPPC:PA domains to nucleate at surface pressures ≤ 1 mN/m, compared to the ~6 mN/m for pure r-DPPC L_C phase at similar temperatures (Figure S1).

However, real alkane chains are not unstructured cylinders and cannot pack at any arbitrary angle defined by the ratio of tailgroup to headgroup area. Fig. S4A shows the characteristic zig-zag shape and dimensions of an all trans carbon chain. The distance between any two carbons in a chain is 0.0254 nm, and the angle between carbon bonds is 109-112° (*55*). This shape complicates the ideal close packing of the chains as there are only certain discrete tilt angles that allow for nesting one zig-zag in the neighboring zig-zag (Figs. S4B, C, D). In a two-dimensional lattice of three-dimensional alkane chains, the packing is more complicated and several close-packing structures of alkanes are known (*55*). However, even considering the various discrete tilt angles allowed for

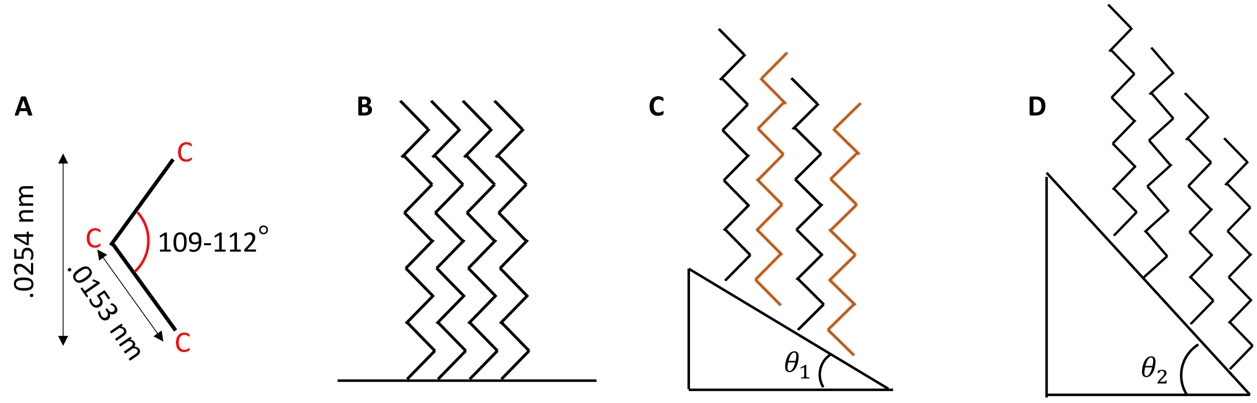

Figure S4. All trans structure of alkanes limits number of close packed tilt angles. A) The all trans configuration of an alkane chain leads to a zig-zag pattern with 0.0254 nm between each two carbon atoms and an angle of 109 – 112° . **B)** Untilted all trans alkanes can pack most tightly if the zig-zag patterns are nested and aligned between molecules. **C)** If the alkane chains are tilted, only discrete tilt angles can align the zig-zags leading to close packing. Here every other chain is rotates so as to offset the chain one "zig". **D)** Here, each chain is offset by a zig-zag leading to a larger tilt, $\theta_2 > \theta_1$. The actual packing motifs of alkanes are further complicated by the real three dimensional shapes of the molecules as described in (55).

all of the classes of packing, an optimal, close-packed crystalline alkane structure may require a fixed mixture of headgroups such as the 2:1 DPPC:HD ratio we find for this system.

Derivation of mass balance for monolayers containing b:1 DPPC:HD + j mol % DChol

Assuming that the co-crystal of DPPC and HD (or PA) grows at a fixed stoichiometric ratio, mole balances can relate the co-crystal stoichiometric ratio, n, with the area fraction of the L_C phase, ϕ_{LC}, as a function of the spreading ratio, *b:1* DPPC:HD. Experimentally, we find that $\phi_{LC}$is identical for r-DPPC:HD, r-DPPC:PA and rac-DPPC:HD at a given surface pressure and temperature for a given spreading solution ratio, but were different for different spreading solution ratios of DPPC to HD or PA. This suggests that there is a fixed ratio of DPPC to HD or PA in the crystal. X-ray diffraction (Fig. 5C) suggests that the lattice spacings are the same for monolayers spread from 2:1 and 3:1 DPPC:HD ratios. Fig. 5B shows that the dihydrocholesterol does not alter the lattice for concentrations from 0 – 8 mol %, so the DChol remains in the L_E phase with any excess DPPC. The molecular areas of the various components in the L_E and L_C phases are well established from isotherms and X-ray diffraction (*7, 52*). $a_{HD} \sim 0.2$ nm^2 is the area/molecule of HD in the crystal; $a_{DC} \sim 0.45$ nm^2 the area/molecule of DPPC in the L_C phase; $a_{DL} \sim$ 0.75 nm^2 the area/molecule of DPPC in the L_E phase; and $a_c \sim 0.25$ nm^2 the area/molecule of DChol in the L_E phase. If the spreading solution contains *N* moles of HD or PA, it contains *bN* moles of DPPC. In our experiments, *b* = 9, 5, 3 and 2 for the various films. The assumed stoichiometric ratio of DPPC to HD (or PA) in the L_C phase crystal is *n*. We assume $b \geq n$ for our calculations. $j = 0.015$ is the mole fraction of DChol in the mixture, which is assumed to reside exclusively in the L_E phase (the calculations do not change much for various values of *j*).

At surface pressures ≤ 6 mN/m where we first observe the fingering instability, we assume that the limiting HD or PA is depleted from the L_E melt, so the total area of the black L_C phase consists of HD and DPPC molecules in their condensed state (Eqn. 3 of text):

$$A_B = Na_{HD} + nNa_{DC} \qquad (3)$$

The remaining L_E phase is made up of the residual DPPC and all the DChol. The 0.75 mol % Texas-Red DHPE also resides in the L_E phase but is not explicitly accounted for; it would simply increase the value of j from .015 to .0225 but does not change the results significantly. The total area of the red L_E phase is:

$$A_R = A_{DL} + A_c \qquad (S8)$$

The area of the DPPC in the L_E phase is:

$$A_{DL} = N(b-n)a_{DL} \qquad (S9)$$

The mole fraction, *j*, of DChol can be written in terms of the numbers of molecules of HD, *N*, DPPC, *bN*, and DChol, N_c, in the spreading solution:

$$j = \frac{N_c}{N + bN + N_c} \qquad (S10)$$

Reorganizing:

$$N_c = \frac{jN(b+1)}{1-j} \qquad (S11)$$

Which gives the DChol area:

$$A_c = \frac{jN(b+1)}{1-j} a_c \qquad (S12)$$

The total area of the red L_E phase is given by (Eqn. 4 of text):

$$A_R = N(b-n)a_{DL} + \frac{j(b+1)Na_c}{1-j} \qquad (4)$$

The LC phase area fraction in the images is:

$$\phi_{LC} = \frac{A_B}{A_B + A_R} \qquad (S13)$$

$$\phi_{LC} = \frac{Na_{HD} + nNa_{DC}}{Na_{HD} + nNa_{DC} + N(b-n)a_{DL} + \frac{j(b+1)Na_c}{1-j}} \qquad (S14)$$

Simplifying, this gives Eqn. 5 of the text:

$$\phi = \frac{a_{HD} + na_{DC}}{a_{HD} + na_{DC} + (b-n)a_{DL} + \frac{j(b+1)a_c}{1-j}} \qquad (5)$$

Eqn. 5 is plotted in Figure 5A for n = 1, 2, 3 as a function of b, the ratio of DPPC to HD (or PA) in the spreading solution. The measured data for all systems is consistent with n = 2. Varying the areas/molecule by 10 % does not make a significant difference in Eqn. 5 nor in the fits to the data in Fig. 5A.

Figure S5 shows representative confocal images (thresholded via ImageJ) of monolayers of b = 2, 3, 5, and 9 to 1 DPPC:HD spreading solutions taken at 5-6 mN/m before the fingering instability. We assumed that the majority of the HD or PA was depleted from the LE phase under these conditions and the domains had the same stoichiometry of n:1 DPPC:HD. The images were thresholded and turned into binary images (black and white) with the black representing the L_C

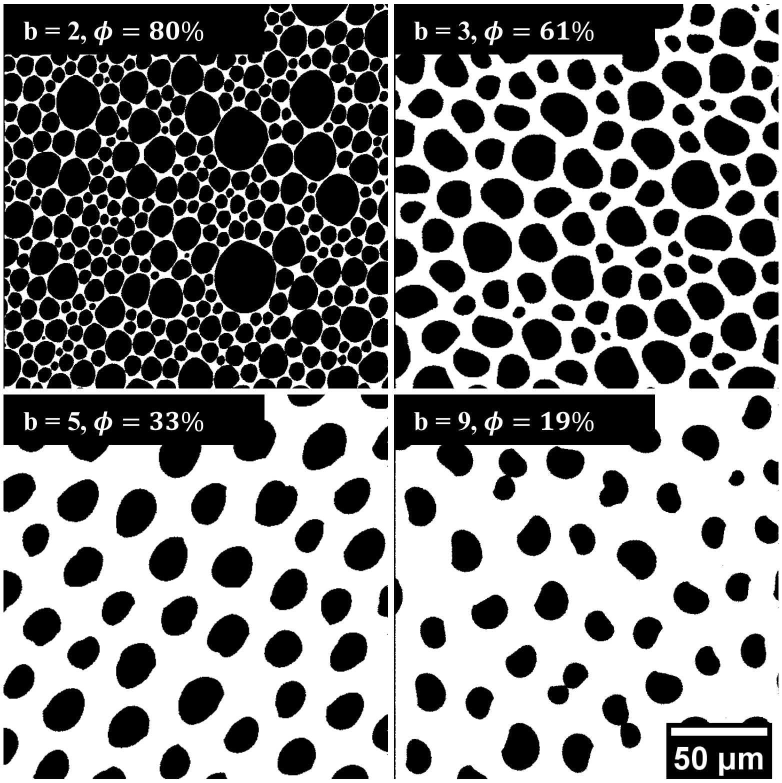

Figure S5. Solid phase area fraction images for b:1 spreading solutions. Confocal images of monolayers of b:1 DPPC:HD spreading solutions for b = 2, 3, 5, and 9. The images are thresholded and turned into binary images (black or white) with the black representing the L_C phase and the white the L_E phase. Image analysis with Image J is used to calculate the fraction of black pixels in each image. In the example images: for b = 2, the fraction of black LC phase $\phi = 0.80$. For b = 3, $\phi = 0.61$, for b = 5, $\phi = 0.33$ and for b = 9, $\phi = 0.19$. From Eqn. 5, analyzing all available images for each composition results in $n = 2.01 \pm 0.2$, or the actual ratio of DPPC to HD or PA in the LC crystals is 2:1. All images are 200×200 μm.

phase and the white the L_E phase. Image J was used to calculate, ϕ, the fraction of black pixels in each image.

Binary DPPC-Dchol nucleation pressures as a function of temperature

After the HD or PA is depleted from the melt, the L_E phase is a mixture of DPPC and DChol. As the surface pressure is increased, DPPC will precipitate in the LC phase depending on the

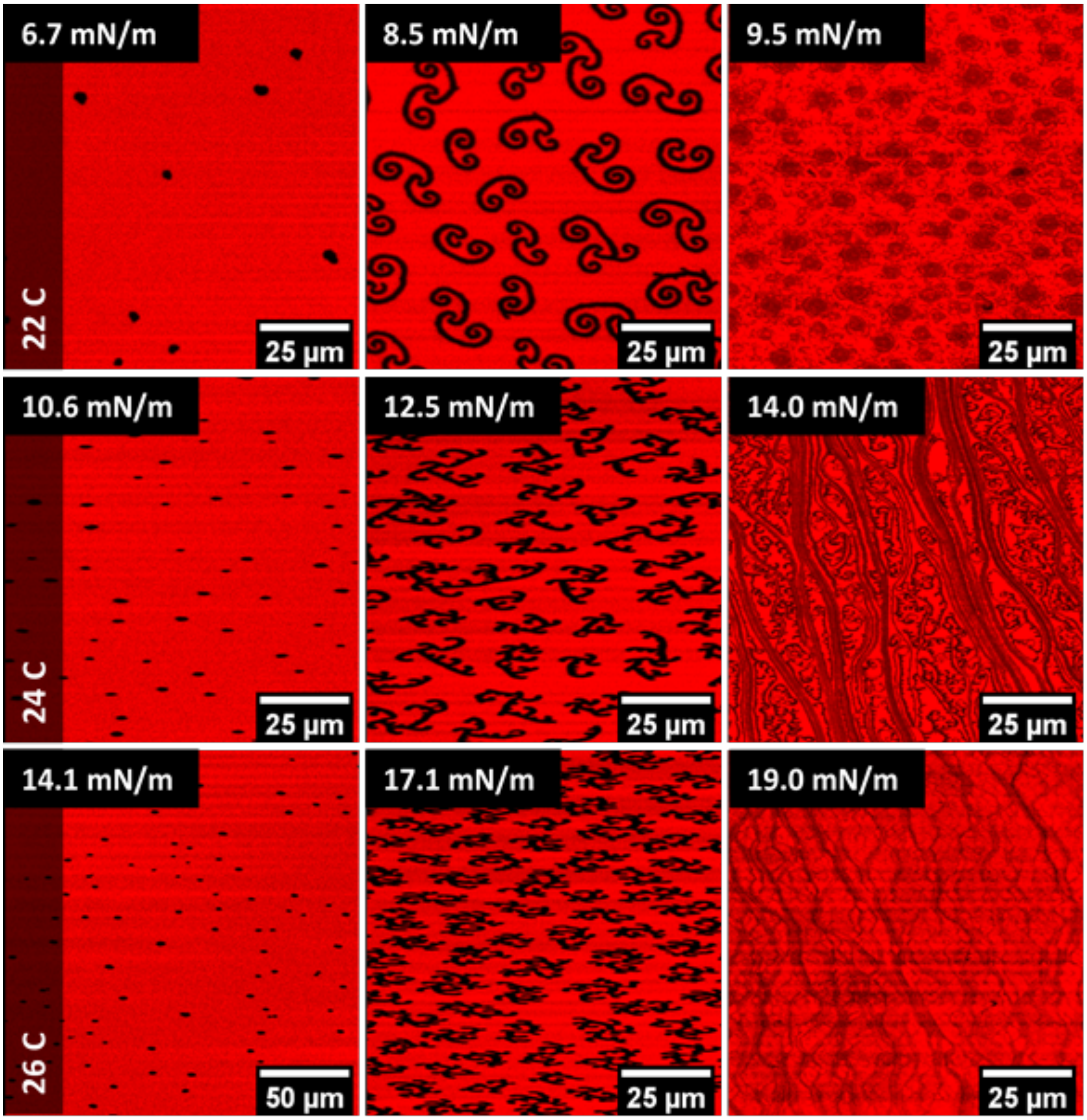

Figure S6. Binary r-DPPC + 1.5 mol % DChol as a function of temperature. As the temperature is increased, the nucleation of the L_C phase (black) occurs at higher surface pressures. As the surface pressure is increased at a given temperature, the domains develop multiple lobes that decrease in width. Within a range of 4-5 mN/m from the start of nucleation, the domain widths reach the resolution limit of our confocal microscope. The crystallization phase diagram (Fig. 6A) of the binary phase (red diamonds) were determined by recording the temperature at which domains first become visible during monolayer compression as a function of subphase temperature.

temperature of the monolayer. Figure S6 shows the nucleation of DPPC domains from a binary monolayer spread from r-DPPC with 1.5 mol % DChol and 0.75 mol % Texas-red DHPE. The onset surface pressure of the L_C phase (black domains) is plotted against temperature in Figure 6A. In the ternary mixture as the surface pressure is increased, the existing domains grow in size and there are no new small domains formed, suggesting that the DPPC grows epitaxially on the original DPPC:HD crystals that formed at lower surface pressures. The transitions surface pressures between the L_E and L_C phases does not change significantly with DChol mole fraction up to 5% as

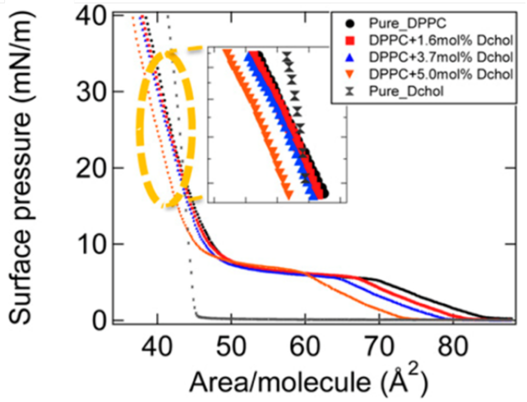

Figure S7. Increasing DChol mol fraction does not alter the coexistence surface pressure. Isotherms of DPPC and various fractions of DChol. The coexistence plateau surface pressure of ~ 7 mN/m is indistinguishable for 0 – 5 mol % DChol at 23 °C. From Ref. 38

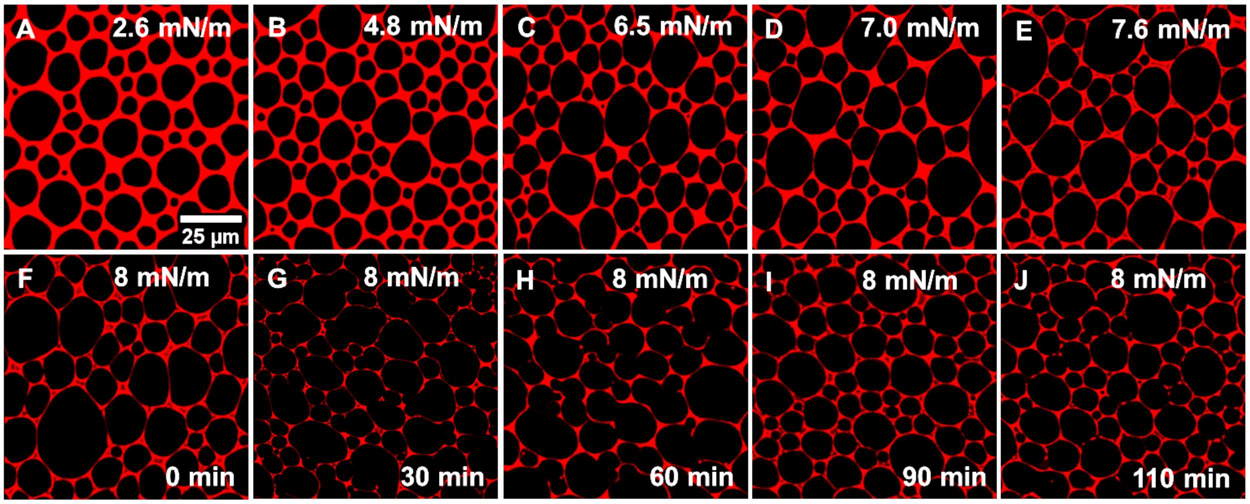

Figure S8. 2:1 r-DPPC:HD + 1.5 mol % DChol does not undergo fingering instability or circle-to-stripe transition. Compression of the 2:1 r-DPPC:HD + 1.5 mol % DChol system does not induce a fingering instability as the surface pressure is increased or a transition to stripes. This is consistent with the hypothesis that the L_C domain composition is fixed at 2:1 DPPC:HD mole ratio. All images are 100 × 100 µm.

shown in the isotherms of Figure S7 (8). For a spreading solution of 2:1 r-DPPC:HD with 1.5 mol % DChol, no fingering instability nor transition to stripes is observed. (Figure S8)

Mullins-Sekerka Instability in Two Dimensions

Although our experiments contain multiple components (DPPC, PA or HD, and DChol), we are primarily interested in the condensation of a DPPC-rich melt (depleted of PA or HD and with trace amounts of DChol) onto a pre-formed crystal of DPPC:PA or DPPC:HD. Neglecting the role of trace DChol on the mass transfer leaves only DPPC to consider. For the purpose of illustration, we thus consider the classical Mullins-Sekerka instability of a pure substance in two dimensions, which directly leads to Eqns. 6, 7 in the main text.

The following derivation is reproduced from Langer (*40, 41*). Consider a two-dimensional, solid crystal occupying the region y < 0, with its boundary along the x-axis. The crystal is growing in the $+y$ direction due to condensation of a liquid melt that originally occupies the region y > 0. The height of the growing crystal is denoted by $y = b(x, t)$. The number of molecules per unit area $\Gamma(x, y, t)$ (in both the solid and liquid phases) obeys the diffusion equation,

$$\frac{\partial \Gamma}{\partial t} = M\nabla^2\mu \qquad (S6)$$

where $\mu(x, y, t)$ is the chemical potential (units of energy per molecule) and M is a mobility (units of squared number of molecules per energy per time). The boundary conditions are

$$\mu|_{y\to\infty} = \mu_{\text{bulk}} \text{ (bulk liquid condition)} \qquad (\text{S7a})$$

$$\mu|_{y=b(x,t)} = \mu_{\text{eq}} - \frac{\lambda}{\Delta\Gamma}\kappa \text{ (Gibbs-Thomson condition)} \qquad (\text{S7b})$$

where λ is the line tension. $\Delta\Gamma = (\Gamma_s - \Gamma_L)$ is the jump in number density across the solid-liquid interface, which can be appreciable in lipid monolayers as $a_{DC} \sim 0.45 \text{ nm}^2 = {}^{1}/_{\Gamma_s}$ is the area/molecule of DPPC in the solid LC phase and $a_{DL} \sim 0.75\ nm^2 = {}^{1}/_{\Gamma_L}$ is the area per molecule in the liquid LE phase. The interfacial boundary curvature, $\kappa = \partial^2 b/\partial x^2/[1 + (\partial b/\partial x)^2]^{3/2}$. The velocity of the interface is given by the jump in diffusive flux across it,

$$v \equiv \frac{\partial b}{\partial t} = \left(\frac{M}{\Delta\Gamma}\right)\left[\left(\frac{\partial \mu}{\partial n}\right)_S - \left(\frac{\partial \mu}{\partial n}\right)_L\right] \text{ (Stefan condition)} \qquad (S8)$$

where we have assumed that the mobility M is the same in both the solid and liquid phases; $\hat{\boldsymbol{n}} = [\hat{\boldsymbol{y}} - (\partial b/\partial x)\hat{\boldsymbol{x}}]/\sqrt{1 + (\partial b/\partial x)^2}$ is the unit normal to the interface (directed from solid to liquid); $\left(\frac{\partial \mu}{\partial n}\right)_S - \left(\frac{\partial \mu}{\partial n}\right)_L = \Delta(\partial \mu/\partial n)$ is the jump in the chemical potential gradient in the normal direction across the solid-liquid interface.

To make further progress, we must relate the number density Γ to the chemical potential μ. If the deviations from equilibrium are small, we can expand the chemical potential of the liquid and solid in Taylor series around the equilibrium state:

$$\tilde{\mu} \equiv \mu - \mu_{\mathrm{eq}} \approx \alpha_L(\Gamma - \Gamma_{\mathrm{L}}), \qquad y > b(x,t) \qquad (S9a)$$

$$\approx \alpha_S(\Gamma - \Gamma_{\mathrm{S}}), \qquad y < b(x,t) \qquad (S9b)$$

with Γ_{L} and Γ_{S} the equilibrium number densities in the liquid and solid, respectively (as in Eqn. S7b.) $\alpha_L \equiv (\partial \mu/\partial \Gamma)_{\Gamma=\Gamma_{\mathrm{L}}}$ and $\alpha_S \equiv (\partial \mu/\partial \Gamma)_{\Gamma=\Gamma_{\mathrm{S}}}$ are the Taylor coefficients in a density expansion about the equilibrium coexistence conditions in the liquid and solid phases. If we further assume that α_L and α_S are independent of position, then the diffusion equation simplifies to

$$\frac{\partial \tilde{\mu}}{\partial t} = D_L \nabla^2 \tilde{\mu}, \qquad y > b(x,t) \qquad (S10a)$$

$$\frac{\partial \tilde{\mu}}{\partial t} = D_S \nabla^2 \tilde{\mu}, \qquad y < b(x,t) \qquad (S10b)$$

where $D_L = \alpha_L M$ and $D_S = \alpha_S M$ are the diffusivities in the liquid and solid phases, respectively (units of area per time).

Let $b_0(t) \equiv \lim_{L\to\infty} \frac{1}{L}\int_{-L}^{L} b(x,t)\,dx$ be the mean height of the interface, which evolves in time. We may define the Lagrangian coordinate $\tilde{y}(y,t) \equiv y - b_0(t)$ and assume that $\tilde{\mu} = \tilde{\mu}[x, \tilde{y}(y,t)]$ depends implicitly on time through $b_0(t)$ (that is, $\tilde{\mu}$ has no explicit time dependence). In a quasi-steady state in a frame that co-moves with the interface, the diffusion equation then becomes

$$D_L \tilde{\nabla}^2 \tilde{\mu} + v_0 \frac{\partial \tilde{\mu}}{\partial \tilde{y}} = 0, \qquad \tilde{y} > b(x,t) - b_0(t) \qquad (S11a).$$

in the liquid

$$D_S \tilde{\nabla}^2 \tilde{\mu} + v_0 \frac{\partial \tilde{\mu}}{\partial \tilde{y}} = 0, \qquad \tilde{y} < b(x,t) - b_0(t) \qquad (S11b)$$

and in the solid where $\tilde{\nabla} \equiv (\partial/\partial x)\hat{\boldsymbol{x}} + (\partial/\partial \tilde{y})\hat{\boldsymbol{y}}$ and $v_0 \equiv db_0/dt$ is the mean velocity of the interface.

In the absence of interfacial fluctuations, the problem is entirely one-dimensional, $\tilde{\mu} = \tilde{\mu}_0(\tilde{y})$, and satisfies

$$D_L \frac{d^2 \tilde{\mu}_0}{d\tilde{y}^2} + v_0 \frac{d\tilde{\mu}_0}{d\tilde{y}} = 0, \qquad \tilde{y} > 0 \qquad (S12a)$$

$$D_S \frac{d^2 \tilde{\mu}_0}{d\tilde{y}^2} + v_0 \frac{d\tilde{\mu}_0}{d\tilde{y}} = 0, \qquad \tilde{y} < 0 \qquad (S12b)$$

with the boundary conditions,

$$\tilde{\mu}_0|_{\tilde{y}\to\infty} = -\alpha_L \Delta\Gamma \qquad (S13a)$$

$$\tilde{\mu}_0|_{\tilde{y}=0} = 0 \qquad (S13b)$$

$$v_0 \equiv \frac{db_0}{dt} = \left(\frac{M}{\Delta\Gamma}\right)\left[\left(\frac{d\tilde{\mu}_0}{d\tilde{y}}\right)_S - \left(\frac{d\tilde{\mu}_0}{d\tilde{y}}\right)_L\right] \qquad S(13c)$$

where we have substituted $\mu_{\text{bulk}} = \mu_{\text{eq}} - \alpha_L \Delta\Gamma$ into the bulk condition (required for self-consistency). The quasi-steady, 1D solution for $\tilde{\mu}_0(\tilde{y})$ is (Figure S9)

$$\tilde{\mu}_0(\tilde{y}) = \alpha_L \Delta\Gamma \left(e^{-\tilde{y}/l_D} - 1\right), \qquad \tilde{y} > 0. \quad (S14)$$

$$= 0, \qquad \tilde{y} < 0$$

where $l_D = D_L/v_0$ is the diffusion length in the melt. The jump in the normal derivative of $\tilde{\mu}_0$ across $\tilde{y} = 0$ is $\Delta(d\tilde{\mu}_0/d\tilde{y}) = \alpha_L \Delta\Gamma / l_D$, which, according to the Stefan condition, gives the interfacial velocity, $v_0 = \alpha_L M / l_D = D_L / l_D$ (an identity). Thus, the solution is self-consistent.

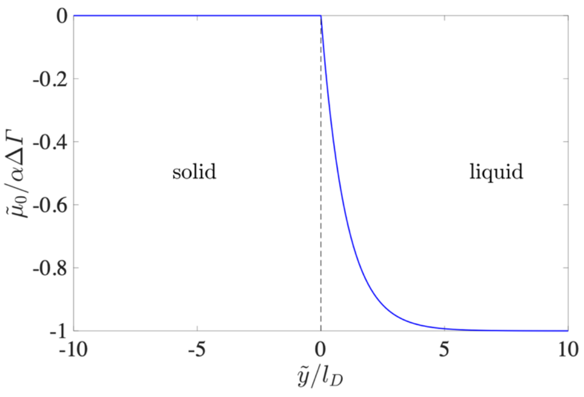

Figure S9. Chemical potential field across a planar solid-liquid interface from Eqn. S14.

We would now like to analyze the stability of the 1D solution above to wave-like interfacial perturbations,

$$b(x,t) = b_0(t) + \epsilon l_D e^{ikx+\omega t} \qquad (S15)$$

or

$$\tilde{b}(x,t) \equiv b(x,t) - b_0(t) = \epsilon l_D e^{ikx+\omega t} \qquad (S16)$$

where k is the wavenumber, ω is the amplification rate, and ϵ is the (small) amplitude of the perturbation. In Eqn. S16, the perturbation is assumed small compared to the diffusion length l_D. Setting $\epsilon = 0$ gives $\tilde{b} = 0$ and we recover our 1D solution, $\tilde{\mu} = \tilde{\mu}_0$, for the chemical potential. For small, but finite ϵ, $\tilde{b} \neq 0$ and $\tilde{\mu}$ is perturbed from this 1D base state. Since ϵ is small, we assume (and verify *a posteriori*) that the chemical potential is weakly perturbed as

$$\tilde{\mu}(x,\tilde{y},t) = \tilde{\mu}_0(\tilde{y}) + \epsilon\tilde{\mu}_k(\tilde{y})e^{ikx+\omega t} \qquad (S17)$$

where $\tilde{\mu}_0$ is given above and $\tilde{\mu}_k$ satisfies (from Eqn. S11)

$$D_L\left(\frac{d^2\tilde{\mu}_k}{d\tilde{y}^2} - k^2\tilde{\mu}_k\right) + v_0\frac{d\tilde{\mu}_k}{d\tilde{y}} = 0, \qquad \tilde{y} > 0 \qquad (S18a)$$

$$D_S\left(\frac{d^2\tilde{\mu}_k}{d\tilde{y}^2} - k^2\tilde{\mu}_k\right) + v_0\frac{d\tilde{\mu}_k}{d\tilde{y}} = 0, \qquad \tilde{y} < 0 \qquad (S18b)$$

The solution for $\tilde{\mu}_k$ is

$$\tilde{\mu}_k = A_L e^{-q\tilde{y}}, \qquad \tilde{y} > 0 \qquad (S19a)$$

$$\tilde{\mu}_k = A_S e^{+q'\tilde{y}}, \qquad \tilde{y} < 0 \qquad (S19b)$$

where q and q' are the positive roots of the quadratic equations ($\beta \equiv D_S/D_L$),

$$q^2 - \frac{q}{l_D} - k^2 = 0, \qquad q'^2 + \frac{q'}{\beta l_D} - k^2 = 0 \qquad (S20)$$

and A_L, A_S are obtained by applying the Gibbs-Thomson condition,

$$\tilde{\mu}|_{\tilde{y}=\tilde{b}(x,t)} = -\frac{\lambda}{\Delta\Gamma}\kappa = -\frac{\lambda}{\Delta\Gamma}\frac{\partial^2\tilde{b}/\partial x^2}{\left[1 + \left(\partial\tilde{b}/\partial x\right)^2\right]^{3/2}} \qquad (S21)$$

Substituting $\tilde{b} = \epsilon l_D e^{ikx+\omega t}$ and $\tilde{\mu} = \tilde{\mu}_0 + \epsilon \tilde{\mu}_k e^{ikx+\omega t}$ into the Gibbs-Thomson condition and linearizing for small ϵ yields a pair of equations for A_L, A_S:

$$A_L - \alpha_L \Delta\Gamma = A_S = -\frac{\lambda l_D}{\Delta\Gamma} k^2 \qquad (S22)$$

These expressions naturally follow from collecting all terms of $O(\epsilon)$ in the Gibbs-Thomson condition. This confirms that the perturbation to $\tilde{\mu}$ is small like $O(\epsilon)$, as was assumed in Eqn. S17.

Applying the Stefan condition gives the velocity of the interface,

$$v = v_0 + \frac{\partial \tilde{b}}{\partial t} = \left(\frac{M}{\Delta\Gamma}\right)\left[\left(\frac{d\tilde{\mu}_0}{d\tilde{y}}\right)_S - \left(\frac{d\tilde{\mu}_0}{d\tilde{y}}\right)_L\right]_{\tilde{y}=\tilde{b}(x,t)}$$

$$= \frac{M}{\Delta\Gamma\sqrt{1+\left(\partial\tilde{b}/\partial x\right)^2}}\left[\left(\frac{\partial\tilde{\mu}}{\partial\tilde{y}} - \frac{\partial\tilde{b}}{\partial x}\frac{\partial\tilde{\mu}}{\partial x}\right)_S - \left(\frac{\partial\tilde{\mu}}{\partial\tilde{y}} - \frac{\partial\tilde{b}}{\partial x}\frac{\partial\tilde{\mu}}{\partial x}\right)_L\right]_{\tilde{y}=\tilde{b}(x,t)} \qquad (S23)$$

where the jump in chemical potential gradient is evaluated at $\tilde{y} = \tilde{b}(x,t)$. Again, substituting for $\tilde{b}$ and $\tilde{\mu}$ and linearizing for small ϵ yields the dispersion relation,

$$l_D\omega + v_0 = \frac{M}{\Delta\Gamma}(A_L q + A_S q') \qquad (S24)$$

Substituting for A_L, A_S, q, and q' and expanding for $kl_D \gg 1$, for which $q, q' \approx k$, then gives (Figure S10):

$$\omega = kv_0(1 - 2l_D l_C k^2) \equiv kv_0(1 - k^2/k_0^2) \qquad (S25)$$

where $l_C = \lambda/\alpha_L(\Delta\Gamma)^2$ is the capillary length and $k_0 = 1/\sqrt{2l_C l_D}$ is the neutral stability wavenumber. Eqn. (S25), which also appears in Langer's paper (41) as Eqn. 3.14, states that the amplitude of the waves grow ($\omega > 0$) when $k < k_0$, where $k_0 = 1/\sqrt{2l_D l_C}$. The fastest growing wavenumber, for which ω is maximized, is $k^* = k_0/\sqrt{3}$ as described in the main text.

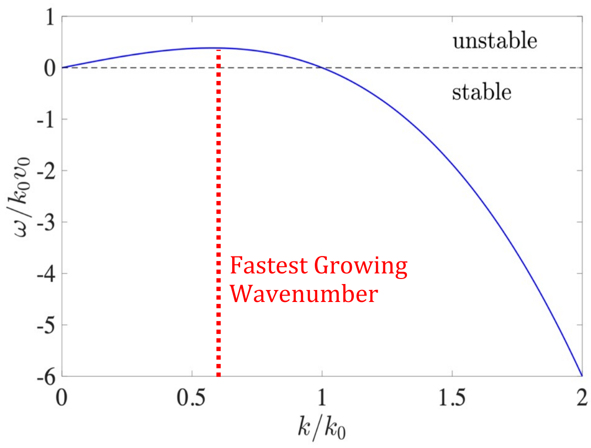

Figure S10. Dispersion relation between the amplification rate and the wavenumber.

The above analysis assumes that the driving force for growth of the solid phase (chemical potential gradient) is due solely to the diffusive flux set up by concentration gradients in the melt phase. It is also possible, though not explicitly considered here, that the dipolar electrostatic force due to the mismatch in dipole moment density across the solid-liquid phases contributes to the chemical potential. As the fingers have a high aspect ratio shape, it is likely that the dipole-dipole repulsion stabilizes the fingering instability and may alter the fastest growing wavenumber.

The values of the convexity, $\alpha_L \equiv (\partial\mu/\partial\Gamma)_{\Gamma=\Gamma_{\mathrm{L}}}$ of the free energy surface can be roughly approximated from the π-A isotherm in the $\mathrm{L_E}$ phase. For an isothermal, 1-component system (neglecting the fact that the monolayer is multicomponent),

$$d\pi = \Gamma d\mu \text{ or } d\mu/d\pi = 1/\Gamma, \qquad \text{(S26)}$$

which is the Gibbs-Duhem relation for a 1 component monolayer. The chain rule gives

$$(\partial\mu/\partial\Gamma)_{\Gamma=\Gamma_{\mathrm{L}}} = (\partial\mu/d\pi)(\partial\pi/d\Gamma) = (1/\Gamma)(\partial\pi/d\Gamma) \qquad (S27)$$

As $\Gamma = 1/\mathrm{A}$ in which A is the area/molecule in an isotherm experiment,

$$\partial\pi/d\Gamma = \partial\pi/d\left(\frac{1}{A}\right) = -A^2\,\partial\pi/dA, \text{ and } (\partial\mu/\partial\Gamma)_{\Gamma=\Gamma_{\mathrm{L}}} = -A^3\,\partial\pi/dA. \qquad (S28)$$

In principle, from the slope of the π-A isotherm at the beginning of the solid-liquid coexistence region, and the area/molecule, A, to estimate α_L. $\Delta\Gamma$ can also be determined from isotherms and X-ray diffraction measurements by knowing A_{LC}, the area per molecule in the $\mathrm{L_C}$ phase and A_{LE} the area per molecule in the $\mathrm{L_E}$ phase at coexistence:

$$\Delta\Gamma = {}^{1}\!/_{A_{LC}} - {}^{1}\!/_{A_{LE}} \qquad (S29)$$

The capillary length can be approximated as:

$$l_C = {}^{\lambda}\!/_{\alpha_L(\Delta\Gamma)^2} \approx {}^{-\lambda}\!\Big/\!\left[(A^3\,\partial\pi/dA)\left({}^{1}\!/_{A_{LC}} - {}^{1}\!/_{A_{LE}}\right)^2\right] \qquad (S30)$$

However, l_C requires knowing the line tension, λ, which we do not have a good way of measuring directly.

The diffusion length, $l_D = D_L/_v$ is somewhat easier to measure from confocal imaging. The diffusivity in the L_E phase can be measured using fluorescence recovery after photobleaching, and v can be estimated from following single domains as they grow to determine the crystal growth velocity. A second theory for fingering by Coriell & Parker (58) suggests that l_D should be replaced by the domain radius, which can be measured directly from the images. Evaluating these parameters is beyond the scope of the current work.

Origin of Theory of the Circle to Stripe Transition

The basic model was developed by McConnell and coworkers (5); details are available in a number of reviews and original papers that appear in the references. The opposing contributions that determine the domain shapes and sizes are the sum of the dipole-dipole electrostatic energy within a domain, F_{el}, and the line tension F_λ.

$$F = F_{el} + F_\lambda \qquad (S31)$$

The electrostatic energy, F_{el} is the sum of the inverse cubic dipole-dipole interactions within each domain:

$$F_{el} = \sum_{k<k'} \mu^2\delta^2/_{r^3_{kk'}} \qquad (S32)$$

in which μ is the density of the dipole moment perpendicular to the monolayer and δ^2 is the area occupied by a given molecular dipole. This result can be cast as a line integral around the perimeter of the domain:

$$F_{el} = -\frac{\mu^2}{2}\oint \frac{dR \cdot dR'}{r} \qquad (S33)$$

in which R and R' are position vectors for the two molecular dipoles of strength μ and r is the distance between the position vectors (18).

$$F_\lambda = \oint \lambda dR \qquad (S34)$$

This line integral formalism allows for a possible variation in λ with position along the boundary. An analogous set of equations was derived by Goldstein and coworkers (12) for ferrofluids in a magnetic field that showed the connection to McConnell's theory for monolayers with dipole density differences and line tension. The theory was expanded to describe domain shape instabilities (12, 27). Eqns. S33 and S34 are simple in principle, but analytic solutions are difficult to find for complex domain geometries. This led to the simplified geometry of squares and rectangles used in the manuscript (17) that provides an analytic solution (Eqns. 8-14 of the main text).

Stripe to circle reversibility with surface pressure

Crossing the binary DPPC-DChol border in Fig. 6A by decreasing the surface pressure at a constant temperature causes the stripes to revert to the asymmetric circular form as shown in Figure

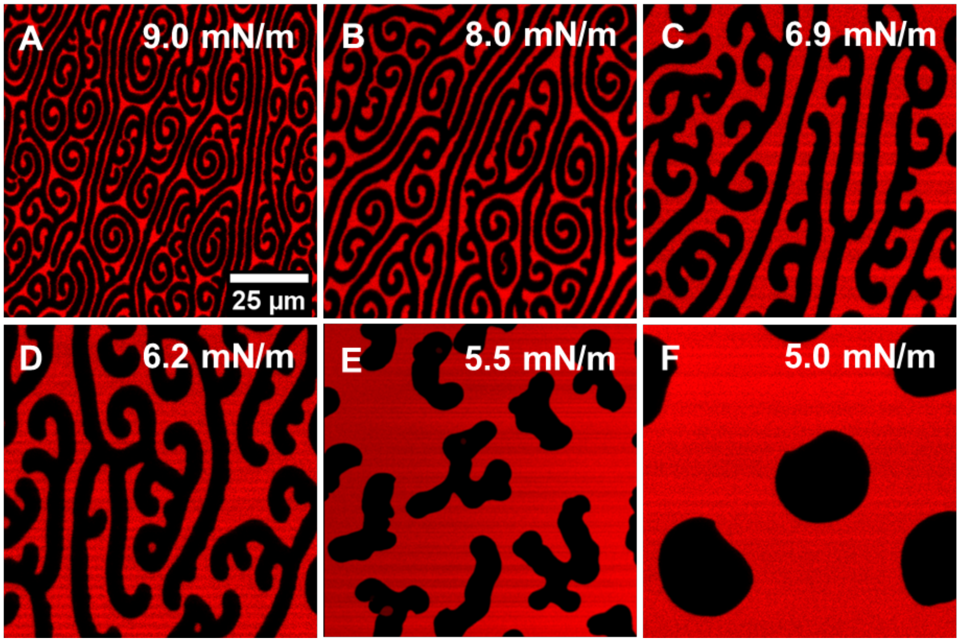

Figure S11. Reversibility via surface pressure at constant temperature. Representative images for 5:1 r-DPPC:HD + 1.5 mol % DChol. The stripe widths are reversible during monolayer expansion (reduction of surface pressure) at constant temperature (22°C). Compare to Fig. 1 (second row) for the circle-to-stripe transition as the surface pressure is increased. Decreasing the surface pressure below the binary phase crystallization conditions (22°C and 6 mN/m) results in stripes reverting back to circles. The time scale for the conversion from stripes to circles depends on the details of the experiment. We find the transition to occur on the order of minutes when the pressure is slightly below the crystallization conditions, and as fast as several seconds when the surface pressure is quickly brought near to 0 mN/m. All images are 100 × 100 µm.

S11. As is the case for the stripe to circle transition induced by increasing temperature at constant surface pressure (Figure 9), the domains do not exhibit a fingering instability during the reverse transition.

Verification of stripe width via image processing

Manual stripe width measurements were complemented by using an image analysis scheme written in Python. First, domains/stripes were identified and analyzed individually. The boundary of the stripe was determined through Canney edge detection, and skeletonization was used to estimate the axis of the stripe. A pruning algorithm was applied to determine the longest axis along a domain. The minimum distance between each point of the axis and the boundary was then measured. Because the domains/stripes are so uniform in width a histogram of these distances is strongly peaked, estimating the half-width of the stripe. An improved approach using the medial axis instead of a skeletonized axis produced similar results.

Replacing Texas Red DHPE by Soluble Rhodamine 123

To ensure that the transition is not influenced by the 0.75 mol % Texas Red DHPE lipid dye that is trapped at the interface, we replaced the lipid dye with water soluble rhodamine 123 in the subphase. We have found that rhodamine in the subphase preferentially adsorbs to disordered regions of the monolayer and is excluded from ordered regions, similar to the water-insoluble lipid

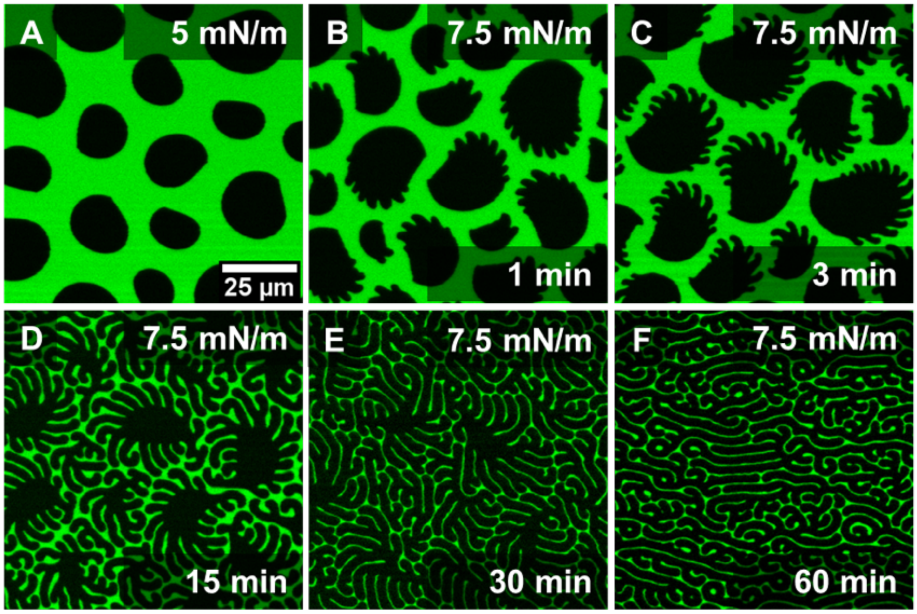

Figure S12. Replacing insoluble fluorescent lipid dye with soluble dye. Replacing the insoluble Texas Red DHPE lipid dye that resides in the monolayer with the green water-soluble Rhodamine 123 does not change the fingering instabilities or the circle to stripe transition. The rhodamine preferentially adsorbs to the L_E phase domains. All images are 100×100 μm

dyes (*59*). Figure S12 shows that the morphological transition is the same for both the Texas Red DHPE and the rhodamine 123.
